# Transcriptional and epigenetic regulators of human CD8^+^ T cell function identified through orthogonal CRISPR screens

**DOI:** 10.1038/s41588-023-01554-0

**Published:** 2023-11-09

**Authors:** Sean R. McCutcheon, Adam M. Swartz, Michael C. Brown, Alejandro Barrera, Christian McRoberts Amador, Keith Siklenka, Lucas Humayun, Maria A. ter Weele, James M. Isaacs, Timothy E. Reddy, Andrew S. Allen, Smita K. Nair, Scott J. Antonia, Charles A. Gersbach

**Affiliations:** 1 Department of Biomedical Engineering, Duke University, Durham, NC USA; 2 Center for Advanced Genomic Technologies, Duke University, Durham, NC USA; 3 Department of Surgery, Duke University Medical Center, Durham, NC USA; 4 Department of Neurosurgery, Duke University School of Medicine, Durham, NC USA; 5 Department of Biostatistics and Bioinformatics, Duke University Medical Center, Durham, NC USA; 6 Department of Pharmacology and Cancer Biology, Durham, NC USA; 7 Duke Cancer Institute Center for Cancer Immunotherapy, Duke University School of Medicine, Durham, NC USA; 8 Department of Pathology, Duke University School of Medicine, Durham, NC USA

**Keywords:** Functional genomics, Immunotherapy, Cancer

## Abstract

Clinical response to adoptive T cell therapies is associated with the transcriptional and epigenetic state of the cell product. Thus, discovery of regulators of T cell gene networks and their corresponding phenotypes has potential to improve T cell therapies. Here we developed pooled, epigenetic CRISPR screening approaches to systematically profile the effects of activating or repressing 120 transcriptional and epigenetic regulators on human CD8^+^ T cell state. We found that BATF3 overexpression promoted specific features of memory T cells and attenuated gene programs associated with cytotoxicity, regulatory T cell function, and exhaustion. Upon chronic antigen stimulation, BATF3 overexpression countered phenotypic and epigenetic signatures of T cell exhaustion. Moreover, BATF3 enhanced the potency of CAR T cells in both in vitro and in vivo tumor models and programmed a transcriptional profile that correlates with positive clinical response to adoptive T cell therapy. Finally, we performed CRISPR knockout screens that defined cofactors and downstream mediators of the BATF3 gene network.

## Abstract (web summary)

CRISPR activation/interference screens identify transcriptional regulators of human CD8^+^ T cells, including BATF3. BATF3 overexpression counteracts T cell exhaustion and enhances cancer immunotherapy in in vivo models.

## Main

Adoptive T cell therapy (ACT) holds tremendous potential for cancer treatment by redirecting T cells to cancer cells via expression of engineered receptors that recognize and bind to tumor-associated antigens. The potency and duration of T cell response are associated with defined T cell subsets, and cell products enriched in stem or memory T cells provide superior tumor control in animal models and in the clinic^1–5^. Consequently, precise regulation or programming of T cell state is a promising approach to improve the therapeutic potential of ACT.

T cell state and function are largely regulated by specific transcription factors (TFs) and epigenetic modifiers that process intrinsic and extrinsic signals into complex and exquisitely tuned gene expression programs. For example, TOX^6–10^ and NFAT^11^ program CD8^+^ T cell exhaustion in the context of chronic antigen exposure. Conversely, T cell function can be enhanced by rewiring transcriptional networks through either enforced expression or genetic deletion of specific TFs and epigenetic modifiers. Ectopic overexpression (OE) of specific TFs such as c-JUN^12^, BATF^13^ and RUNX3 (ref. ^14^) or genetic deletion of NR4A^15^, FLI1 (ref. ^16^), members of the BAF chromatin remodeling complex^17,18^, and regulators of DNA methylation^19,20^ can alter T cell state and improve T cell function through diverse mechanisms.

Large-scale CRISPR knockout (CRISPRko)^21–23^ and open reading frame (ORF) OE^24^ screens have further accelerated gene discovery. Compared to these screening modes, it has been more challenging to conduct gene activation or repression screens via epigenome editing in primary human T cells^25^. One study optimized lentiviral production to overcome limitations of delivering large CRISPR-based epigenome editors and then conducted proof-of-concept gene silencing or activation screens to define regulators of cytokine production^25^. However, there remains an expansive opportunity to discover modulators of other T cell states, as well as combinatorial perturbations to dissect gene interactions that control human T cell phenotypes.

In this Article, we developed an approach for CRISPR interference (CRISPRi) or CRISPR activation (CRISPRa) screens in primary human T cells and applied it to systematically profile the effects of 120 genes on human CD8^+^ T cell state. These screens and subsequent characterization revealed that overexpressing BATF3 supports specific features of memory T cells, counters T cell exhaustion and improves tumor control. We conducted pooled CRISPRko screens of all human transcription factor genes (TFome) with or without BATF3 OE to define cofactors and downstream targets of BATF3. More generally, we developed orthogonal CRISPR-based screening approaches to systematically discover regulators of gene networks and complex T cell phenotypes, which should accelerate efforts to engineer T cells with enhanced durability and therapeutic potential.

## Results

### Developing an epigenetic screening platform in human T cells

**Extended Data Fig. 1:**
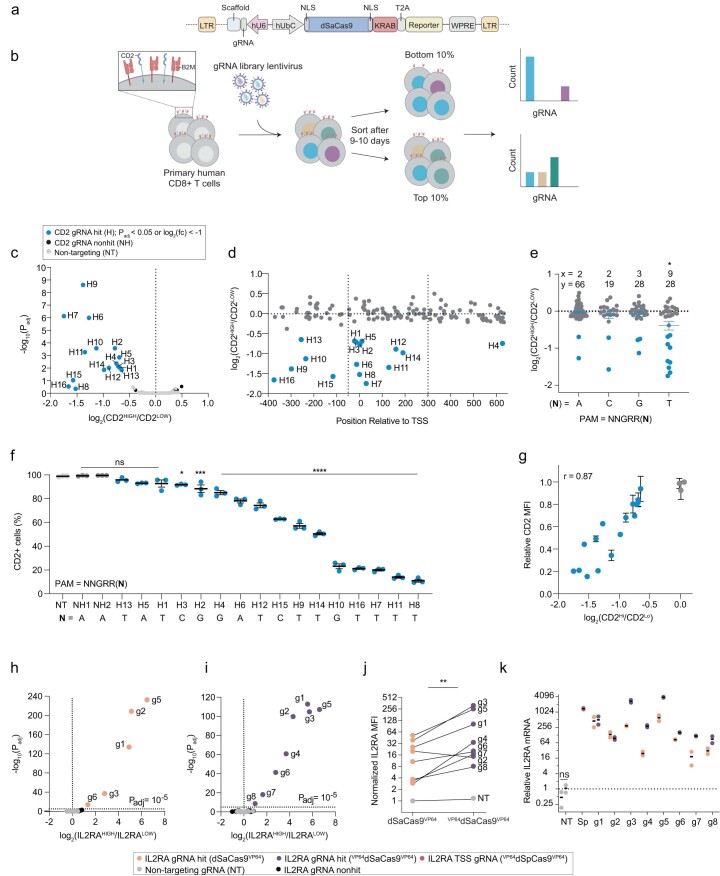
dSaCas9-based epigenetic screening platform. **(a)** Schematic of CRISPRi lentiviral plasmid. **(b)** Schematic of CRISPRi screens in human CD8+ T cells. **(c)** Significance (P_adj_) versus fold change in gRNA abundance between CD2^HIGH^ and CD2^LOW^ populations for CD2 CRISPRi screen. gRNA enrichment was defined using a paired two-tailed DESeq2 test with Benjamini-Hochberg correction. **(d)** CD2 gRNA fold change versus gRNA position relative to TSS. Dashed lines represent previously defined optimal CRISPRi window^32^. **(e)** CD2 gRNA fold change as a function of the final base pair of the PAM. x represents the number of gRNA hits and y represents the total number of gRNAs for each PAM variant. A one-way ANOVA with Dunnett’s post hoc test was used to compare fold change of gRNAs for each PAM variant to NNGRRT (mean values +/− SEM, T versus A (P_adj_ = 0.0003), T versus C (P_adj_ = 0.0399), and T versus G (P_adj_ = 0.0088). **(f)** CD2 gRNA activity plotted in rank order (n = 3 replicates of CD8+ T cells from pooled PBMC donors, mean values +/− SEM). A one-way ANOVA with Dunnett’s post hoc test was used to compare each gRNA to NT. Final base pair of PAM for each gRNA is indicated beneath gRNA label. **(g)** Relationship between CD2 gRNA activity and fold enrichment in screen (n = 18 CD2-targeting gRNAs (16 hits and 2 non-hits) and 1 non-targeting gRNA, mean values +/− SEM with Pearson’s correlation coefficient (r)). Significance (P_adj_) versus fold change in gRNA abundance between IL2RA^HIGH^ and IL2RA^LOW^ populations for the IL2RA CRISPRa Jurkat screens (n = 3 replicates) with **(h)** dSaCas9^VP64^ and **(i)** ^VP64^dSaCas9^VP64^. gRNA enrichment was defined using a paired two-tailed DESeq2 test with Benjamini-Hochberg correction. **(j)** Normalized IL2RA MFI of dSaCas9^VP64^ and ^VP64^dSaCas9^VP64^ Jurkat lines transduced with indicated gRNAs (n = 2 replicates). A two-tailed paired ratio t-test (p = 0.0068) was used to compare gRNA activity between dSaCas9^VP64^ and ^VP64^dSaCas9^VP64^ Jurkat lines. **(k)** Relative IL2RA mRNA expression of Jurkat CRISPRa lines transduced with indicated gRNA on day 9 post-transduction (n = 2, mean values +/− SEM).

**Extended Data Fig. 2:**
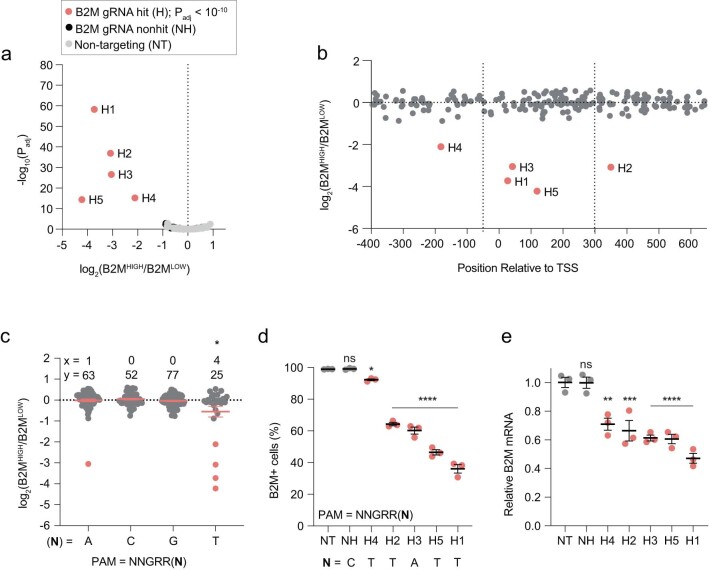
B2M promoter tiling CRISPRi screen in primary human CD8+ T cells. **(a**) Significance (P_adj_) versus fold change in gRNA abundance between B2M^HIGH^ and B2M^LOW^ populations for B2M CRISPRi screen. gRNA enrichment was defined using a paired two-tailed DESeq2 test with Benjamini-Hochberg correction. **(b)** B2M gRNA fold change versus gRNA position relative to TSS. Dashed lines represent previously defined optimal CRISPRi window^32^. **(c)** B2M gRNA fold change as a function of the final base pair of the PAM. x represents the number of gRNA hits and y represents the total number of gRNAs for each PAM variant. A global one-way ANOVA with Dunnett’s post hoc test was used to compare the fold change of gRNAs for each PAM variant to NNGRRT (mean values +/− SEM, T versus A (P_adj_ = 0.002), T versus C (P_adj_ < 0.0001), and T versus G (P_adj_ = 0.0003). **(d)** B2M gRNA activity plotted in rank order (n = 3 replicates of CD8+ T cells from pooled PBMC donors, mean values +/− SEM). A one-way ANOVA with Dunnett’s post hoc test was used to compare each gRNA to NT. Final base pair of PAM for each gRNA is indicated beneath gRNA label. **(e)** Relative B2M mRNA expression of CD8+ cells transduced with indicated gRNA on day 9 post-transduction (n = 3, mean values +/− SEM). A one-way ANOVA with Dunnett’s post hoc test was used to compare each gRNA to NT.

**Extended Data Fig. 3:**
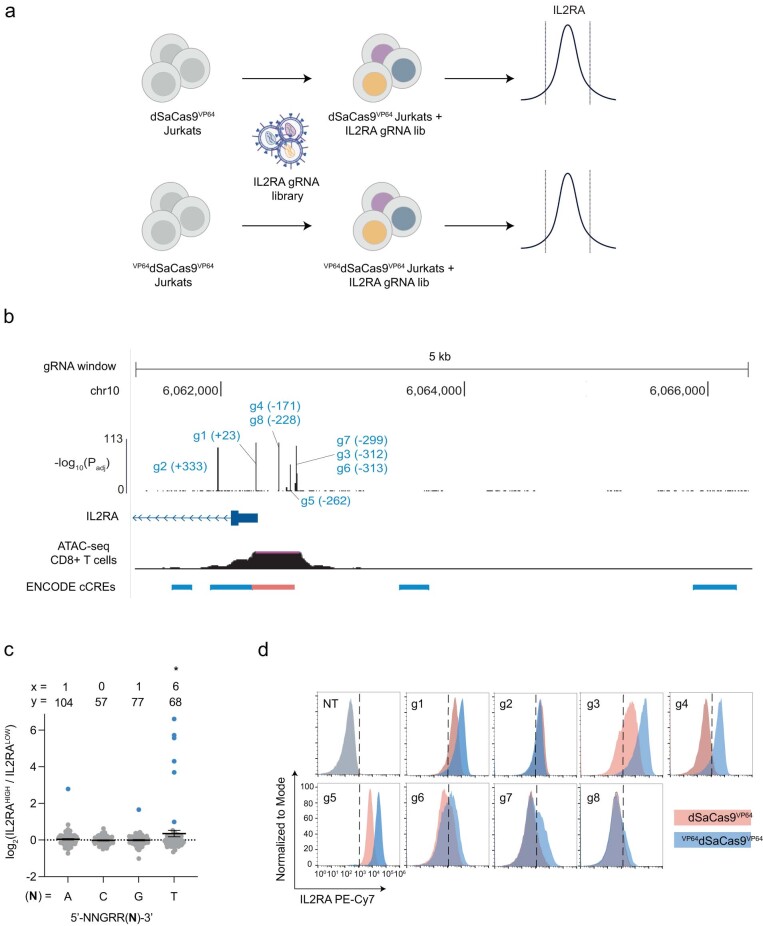
dSaCas9^VP64^ and ^VP64^dSaCas9^VP64^ IL2RA promoter tiling CRISPRa screens in Jurkats. **(a)** Schematic of dSaCas9^VP64^ and ^VP64^dSaCas9^VP64^ IL2RA promoter tiling CRISPRa screens in Jurkats. **(b**) UCSC genome browser track of IL2RA locus with statistical significance displayed for each gRNA in ^VP64^dSaCas9^VP64^ CRISPRa screen. gRNA hits are annotated and labeled in blue. ATAC-seq and ENCODE candidate cis regulatory elements (cCREs) tracks are overlayed for visualization of chromatin accessibility and annotations. cCREs in red are defined as promoter-like elements and cCREs in blue are defined as enhancer-like elements. **(c)** Fold change in IL2RA gRNA abundance as a function of the final base pair of the PAM. x represents the number of gRNA hits and y represents the total number of gRNAs for each PAM variant. A global one-way ANOVA with Dunnett’s post hoc test was used to compare the fold change of gRNAs for each PAM variant to NNGRRT (mean values +/− SEM, T versus A (P_adj_ = 0.0169), T versus C (P_adj_ = 0.0131), and T versus G (P_adj_ = 0.0079). **(d)** Representative overlayed histograms of IL2RA expression for dSaCas9^VP64^ and ^VP64^dSaCas9^VP64^ Jurkat lines on day 9 post-transduction across gRNAs.

*Staphylococcus aureus* Cas9 (SaCas9) has been extensively used for genome editing in vivo as its compact size (3,159 bp) relative to the conventional *Streptococcus pyogenes* Cas9 (SpCas9) enables packaging into adeno-associated virus^26–28^. However, SaCas9 has not been widely used for targeted gene regulation^29,30^ or in the context of an epigenome editing screen. To facilitate delivery to human T cells, we rigorously characterized the activity of dSaCas9 as a repressor or activator using several promoter tiling guide RNA (gRNA) screens in both primary human T cells and the Jurkat cell line (Extended Data Figs. 1–3, Supplementary Fig. 1 and Supplementary Note 1). Collectively, this work demonstrated that dSaCas9 can potently silence or activate target gene expression and informed gRNA design rules.

### CRISPRi/a screens identify regulators of human T cell state

**Fig. 1:**
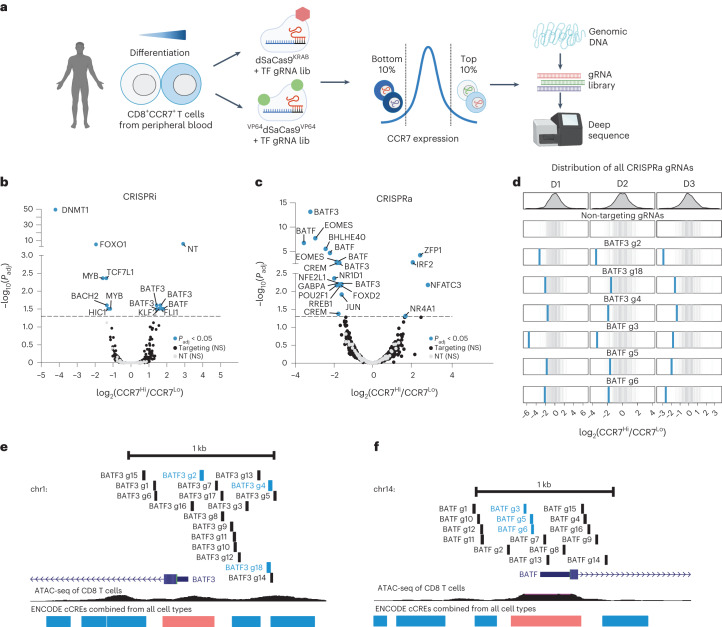
CRISPR interference or activation genetic screens identify transcriptional and epigenetic regulators of human CD8^+^ T cell state. **a**, Schematic of CRISPRi/a screens with TF gRNA library (lib). **b**,**c**, Significance (*P*_adj_) versus fold change in gRNA abundance between CCR7^HIGH^ and CCR7^LOW^ populations for CRISPRi (**b**) and CRISPRa (**c**) screens. gRNA enrichment was defined using a paired two-tailed DESeq2 test with Benjamini–Hochberg correction. **d**, Fold change of BATF3 and BATF CRISPRa gRNA hits for each donor (D1-D3). Blue lines represent BATF3 or BATF gRNAs and gray lines represent the distribution of 120 non-targeting (NT) control gRNAs. **e**,**f**, All BATF3 (**e**) and BATF (**f**) CRISPRa gRNAs in gRNA library relative to TSS, chromatin accessibility and ENCODE candidate *cis*-regulatory elements (cCREs). Blue and black lines represent gRNA hits and nonsignificant gRNAs, respectively. cCRE tracks are overlaid for visualization of promoter-like elements (red) and enhancer-like elements (blue).

We sought to interrogate the effects of repressing or activating genes encoding TFs and epigenetic modifiers on T cell state. We designed a gRNA library targeting 120 TFs and epigenetic modifiers associated with T cell state (Supplementary Fig. 3 and Supplementary Note 2). To detect whether specific gene perturbations altered T cell state, we used CCR7 expression as our screen readout (Fig. 1a and Supplementary Fig. 4). CCR7 is a well-characterized T cell marker and is highly expressed in specific T cell subsets such as naive, stem-cell memory and central memory T cells^31^. We hypothesized it would enable us to capture more subtle changes in T cell state than phenotypic readouts such as proliferation or cytokine production.

The CRISPRi screen recovered many canonical regulators of memory T cells including *FOXO1* (ref. ^32^), *MYB*^33^ and *BACH2* (ref. ^34^)—all of which when silenced led to reduced expression of CCR7, indicative of T cell differentiation towards effector T cells (Fig. 1b and Supplementary Fig. 5a). Interestingly, the most significant hit from the CRISPRi screen was the gene encoding the maintenance DNA methyltransferase *DNMT1*. Genetic disruption of both *TET2* and *DNMT3A*, which encode for proteins that regulate DNA methylation in opposite directions, can improve the therapeutic potential of T cells^19,20^. There was a single nontargeting (NT) gRNA (1/120) hit in the CRISPRi screen. The same NT gRNA emerged as a hit in multiple screens using CCR7 as the readout, suggesting a real off-target effect.

The CRISPRa screen also identified several TFs that have been implicated in CD8^+^ T cell differentiation and function such as *EOMES*^35^, *BATF*^13^ and *JUN*^12^ (Fig. 1c). Importantly, gRNA enrichment was consistent across the three donors and not a function of the number of gRNAs targeting each gene (Fig. 1d and Supplementary Fig. 5). Multiple gRNAs targeting *BATF* and *BATF3* were enriched in reciprocal directions across CRISPRi and CRISPRa screens, and BATF and BATF3 were among the top hits in gene-level analyses, highlighting the power of coupling loss- or gain-of-function perturbations (Supplementary Table 2). The *BATF* and *BATF3* gRNA hits in the CRISPRa screen generally colocalized to regions upstream of the promoter and near the summits of accessible chromatin (Fig. 1e,f).

### scRNA-seq characterization of transcriptional regulators

We next characterized the transcriptomic effects of each candidate gene identified from our CRISPRi or CRISPRa screens using single-cell RNA sequencing (scRNA-seq). We cloned the union set of gRNA hits across CRISPRi/a screens (32 gRNAs) and 8 NT gRNAs into both CRISPRi and CRISPRa plasmids (Supplementary Table 3). We then followed the same workflow as the sort-based screens, but instead of sorting the cells based on CCR7 expression, we profiled the transcriptomes and gRNA identity of ~60,000 cells across three donors for each screen. We aggregated the cells based on gRNA assignment and compared the transcriptional profile of cells with the same gRNA to nonperturbed cells (Supplementary Fig. 6 and Supplementary Note 3).

**Fig. 2:**
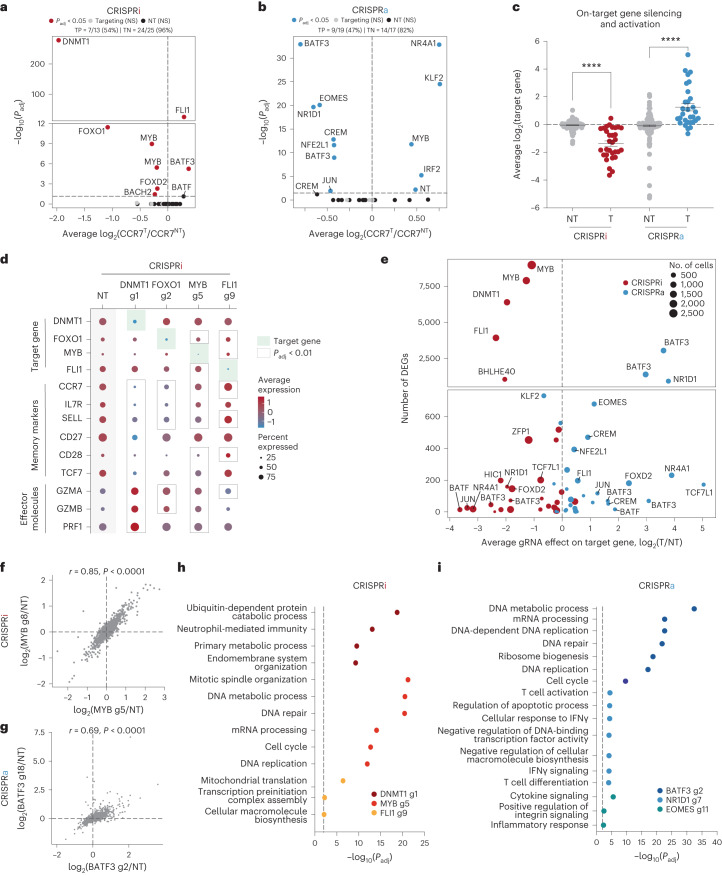
scRNA-seq characterization of candidate genes. **a**,**b**, Significance (*P*_adj_) versus average fold change of CCR7 expression for each gRNA compared to nonperturbed cells for CRISPRi (**a**) and CRISPRa (**b**) perturbations. Significant gRNA effects on CCR7 expression were defined using a two-tailed MAST test with Bonferroni correction. True positive (TP) and negative rates (TN) are displayed above each volcano plot. **c**, Fold change in target gene expression for NT gRNAs and targeting gRNAs across CRISPRi (*n* = 31 gRNAs) and CRISPRa (*n* = 30 gRNAs) perturbations (mean values ± s.e.m.). A two-way ANOVA with Tukey’s post hoc test was used to compare groups. **d**, Dot plot with average expression and percentage of cells expressing target genes, memory markers and effector molecules for the indicated CRISPRi perturbations. Significant gRNA–gene links were defined using a two-tailed MAST test with Bonferroni correction. **e**, Number of DEGs (*P*_adj_ < 0.01) associated with each gRNA versus the gRNA effect on the target gene for both CRISPRi and CRISPRa perturbations. **f**,**g**, Significant gRNA–gene links were defined using a two-tailed MAST test with Bonferroni correction. Correlation of the union set of DEGs between the top two CRISPRi *MYB* gRNAs (**f**) and CRISPRa *BATF3* gRNAs (**g**). Pearson’s correlation coefficient was calculated and then a two-tailed *t*-test was conducted to determine whether the relationship was significant. **h**,**i**, Representative enriched pathways for the top three CRISPRi (**h**) and CRISPRa gRNAs (**i**). Statistical significance was defined using a two-tailed Fisher’s exact test followed by Benjamini–Hochberg correction.

First, we focused on CCR7 expression to validate the results from our CRISPRi/a screens (Fig. 2a,b). Roughly half of the gRNA hits affected CCR7 expression, and the rank order was similar to the sort-based screens. For example, both assays informed that targeted silencing of *DNMT1* or *FOXO1* drastically reduced CCR7 expression levels, which was further confirmed through individual gRNA validations (Supplementary Fig. 7a,b). The gRNA hits that did not validate in the scRNA-seq characterization were represented by fewer cells than validated gRNAs, reaffirming that higher gRNA coverage helps to resolve more subtle changes in gene expression^36^ (Supplementary Fig. 7c and Supplementary Note 4). In addition to confirming gRNA effects on CCR7 expression, the true negative rates were high for both CRISPRi (96%) and CRISPRa (82%), demonstrating the specificity of these sort-based screens (Fig. 2a,b).

We next measured on-target gene silencing or activation. Of gRNAs assigned to at least five cells in each of the CRISPRa and CRISPRi screens, 56/61 gRNAs (92%) silenced or activated their gene target (Fig. 2c). Given that CCR7 was selected as a surrogate marker for a memory T cell phenotype, we expected some perturbations to regulate subset-defining gene expression programs. Indeed, scRNA-seq revealed that silencing the top predicted positive regulators of memory (*DNMT1*, *FOXO1* and *MYB*) led to decreased expression of CCR7 and other memory-associated genes (such as *IL7R*, *SELL*, *CD27*, *CD28* and *TCF7*) and increased expression of effector-associated genes (*GZMA*, *GZMB* and *PRF1*) (Fig. 2d).

**Extended Data Fig. 4:**
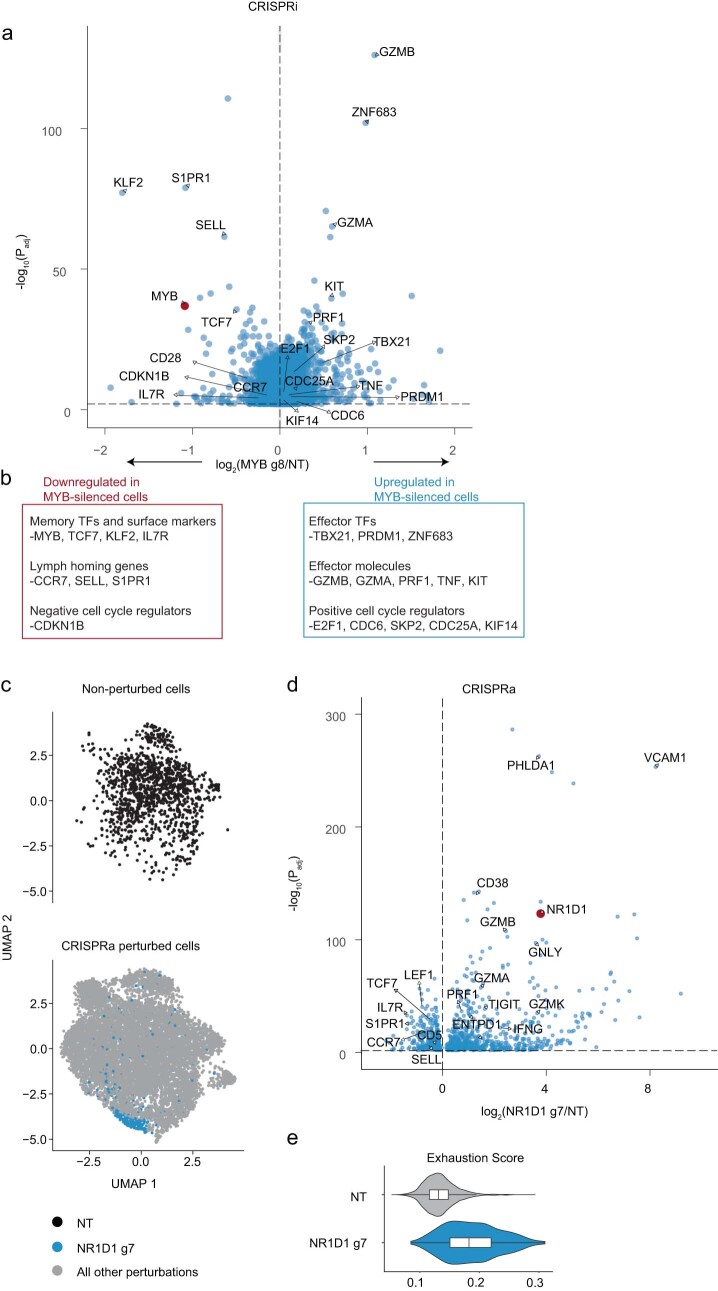
MYB silencing drives T cells towards an effector phenotype and NR1D1 activation induces an exhaustion phenotype. **(a)** Statistical significance (P_adj_) for each gene versus the fold change in gene expression in MYB CRISPRi-perturbed cells relative to non-perturbed cells. Only DEGs (P_adj_ < 0.01, all labeled blue except MYB) are displayed. DEGs were defined using a two-tailed MAST test with Bonferroni correction. **(b)** Classification of annotated DEGs based on their functional role. **(c)** UMAP plot of CRISPRa scRNA-seq characterization with cells split by perturbation status: non-perturbed (top) and perturbed (bottom). Blue data points indicate cells with a NR1D1 gRNA. Cells were clustered using Seurat’s CalcPerturbSig function to mitigate confounding sources of variation such as the donor and phase of cell cycle. **(d)** Statistical significance (P_adj_) for each gene versus the fold change in gene expression in NR1D1 CRISPRa-perturbed cells relative to non-perturbed cells. Only DEGs (P_adj_ < 0.01, all labeled blue except NR1D1) are displayed. DEGs were defined using a two-tailed MAST test with Bonferroni correction. **(e)** Violin plot of exhaustion gene signature score across non-perturbed (n = 2,980 cells) and NR1D1-perturbed (n = 456 cells) in the CRISPRa scRNA-seq screen. Boxplots extend from the lower whisker (minimum value within 1.5 IQR of the first quartile) to the upper whisker (maximum value within 1.5 IQR of the third quartile). The boxed lines represent the first quartile, median, and third quartile. UCell gene signature scores are based on the Mann-Whitney U statistic.

Finally, we examined all differentially expressed genes (DEGs) associated with each perturbation. Endogenous regulation of several TFs and epigenetic-modifying proteins had widespread transcriptional effects with six gene perturbations (four CRISPRi gene perturbations and two CRISPRa gene perturbations) altering expression of >1,000 genes (Fig. 2e). Interestingly, *MYB* repression with two unique gRNAs led to widespread and concordant gene expression changes with 8,976 and 7,899 DEGs (Fig. 2e,f). *MYB* silencing drove a transcriptional program with hallmark features of effector T cells, suggesting that MYB plays a key role in regulating T cell stemness in human CD8^+^ T cells as previously reported in mouse CD8^+^ T cells^33^ (Extended Data Fig. 4a,b and Supplementary Note 5).

Endogenous activation of several TFs including *NR1D1*, *EOMES* and *BATF3* had pronounced effects on T cell state (Fig. 2e,i). Perturbation-driven single-cell clustering revealed a distinct cell cluster with NR1D1 activation that was markedly enriched for exhaustion-associated genes compared to nonperturbed cells (Extended Data Fig. 4c–e and Supplementary Note 6). Notably, a pair of highly concordant *BATF3* gRNAs had the strongest effects among CRISPRa perturbations with 3,056 and 1,402 DEGs (Fig. 2e,g). Gene Ontology analyses revealed that BATF3-induced genes were enriched for DNA and messenger RNA metabolic processing, ribosomal biogenesis and cell-cycle pathways, suggesting an improvement in T cell fitness (Fig. 2i).

### BATF3 OE programs features of memory T cells

**Extended Data Fig. 5:**
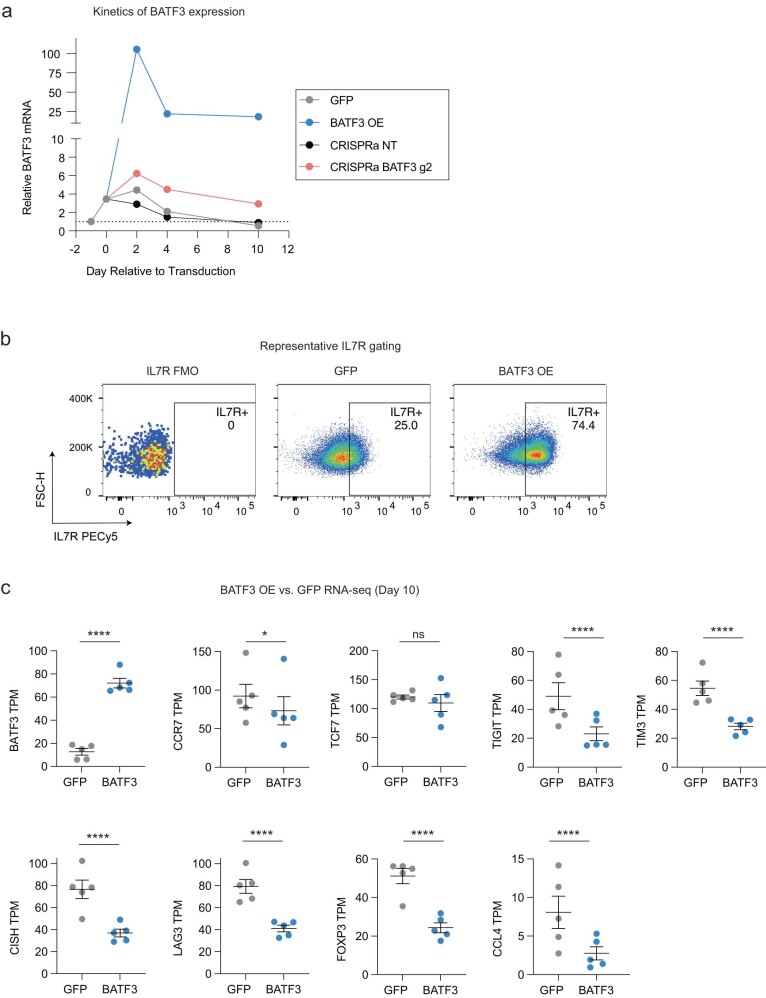
Kinetics of BATF3 expression and effects of BATF3 OE. **(a)** Median BATF3 expression over time relative to baseline expression before T cell activation across groups (n = 3 donors, fold change in BATF3 expression was calculated using 2^−dCT^ method relative to baseline BATF3 expression, internal householding control was excluded because T cell stimulation dramatically alters expression of householding genes such as GAPDH and TBP, input mass of RNA into the reverse transcription reaction was the same for all samples). **(b)** An IL7R fluorescent minus one (FMO, left) control was used to set the IL7R+ gate. Representative IL7R expression of CD8+ T cells from a donor transduced with either GFP (middle) or BATF3 OE (right) on day 8 post-transduction. **(c)** Transcripts per million (TPM) of selected genes: BATF3 (P_adj_ = 1e-7), CCR7 (P_adj_ = 0.01), TCF7 (ns), TIGIT (P_adj_ = 3e-18), TIM3 (P_adj_ =1e-10), CISH (P_adj_ = 6e-11), LAG3 (P_adj_ = 1e-14), FOXP3 (P_adj_ =5e-13), and CCL4 (P_adj_ = 5e-6) with either GFP or BATF3 OE on day 10 post transduction (n = 5 donors, mean values +/− SEM). P_adj_ values were determined using a paired two-tailed DESeq2 test with Benjamini-Hochberg correction.

BATF3 promotes survival and memory formation in mouse CD8^+^ T cells. However, molecular and phenotypic effects of BATF3 in human CD8^+^ T cells have not been well defined^37^. Given that *BATF3* ORF delivery led to higher expression of *BATF3* than endogenous *BATF3* activation (Extended Data Fig. 5a and Supplementary Note 7) and the compact size of the *BATF3* ORF (only 381 bp), we used ectopic BATF3 expression for all subsequent assays and GFP OE as a negative control.

**Fig. 3:**
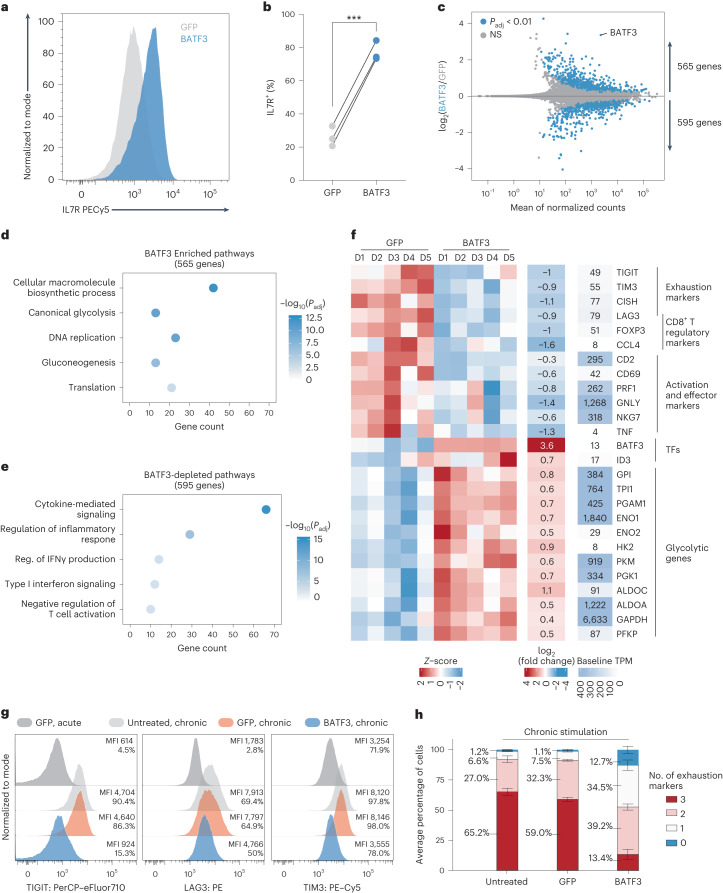
BATF3 OE promotes specific features of memory T cells and counters exhaustion and cytotoxic gene signatures. **a**, Representative histogram of IL7R expression in CD8^+^ T cells with BATF3 OE or control GFP OE on day 8 post-transduction. **b**, Summary statistics of IL7R expression with or without BATF3 OE (*n* = 3 donors with lines connecting the same donor, a two-tailed paired *t*-test (*P* = 0.0004) was used to compare IL7R expression between groups). **c**, Differential gene expression analysis between CD8^+^ T cells with or without BATF3 OE on day 10 post transduction (*n* = 5 donors). DEGs were defined using a paired two-tailed DESeq2 test with Benjamini–Hochberg correction. **d**,**e**, Selected enriched (**d**) and depleted (**e**) biological processes from BATF3 OE. Statistical significance was defined using a two-tailed Fisher’s exact test followed by Benjamini–Hochberg correction. **f**, Heatmap of DEGs (*P*_adj_ < 0.01, *n* = 5 donors) related to T cell exhaustion, regulatory function, cytotoxicity, transcriptional activity and glycolysis. **g**, Representative histograms of exhaustion markers (TIGIT, LAG3 and TIM3) on day 12 after acute or chronic stimulation across groups. **h**, Stacked bar chart with average percentage of CD8^+^ T cells positive for zero, one, two or three exhaustion markers (TIGIT, LAG3 and TIM3) on day 12 after chronic stimulation across groups (*n* = 3 donors, mean values ± s.e.m.).

BATF3 OE markedly increased expression of IL7R, a surface marker associated with T cell survival, long-term persistence and positive clinical response to ACT^38^ (Fig. 3a,b and Extended Data Fig. 5b). We performed RNA-seq across CD8^+^ T cells from five donors to gain an unbiased view of the transcriptomic changes induced by BATF3 OE. Compared to control cells, there were over 1,100 DEGs distributed almost equally between upregulated and downregulated genes (Fig. 3c). Gene Ontology analyses revealed that BATF3 OE increased expression of genes involved in metabolic pathways such as glycolysis and gluconeogenesis, DNA replication and translation (Fig. 3d and Supplementary Table 4).

In contrast, BATF3 OE dampened T cell effector programs and downregulated activation markers, inflammatory cytokines and cytotoxic molecules (Fig. 3e,f). Additionally, BATF3 OE reduced expression of several markers associated with FOXP3^+^ regulatory T cells (T_regs_), which are associated with poor response to ACT^38^. A subset of CD8^+^FOXP3^+^LAG3^+^ T_regs_ suppress T cell activity by secreting CC chemokine ligand 4 (CCL4)^39^. Interestingly, BATF3 OE reduced expression of FOXP3, LAG3 and CCL4 in CD8^+^ T cells (Fig. 3f and Extended Data Fig. 5c).

**Extended Data Fig. 6:**
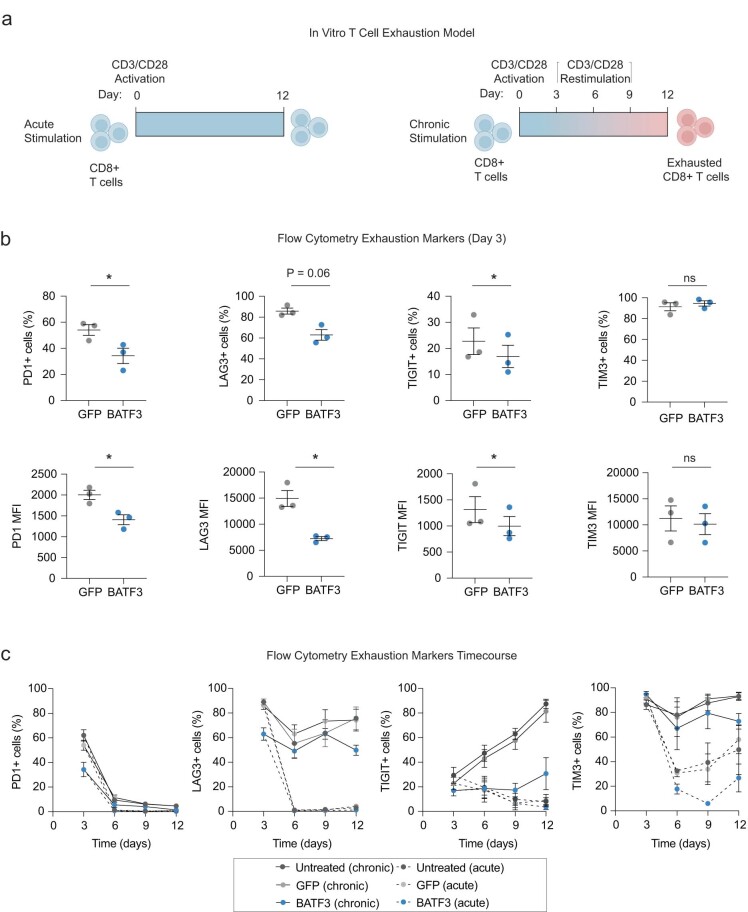
BATF3 OE attenuates expression of T cell exhaustion markers. **(a)** Schematic of acute (left) and chronic stimulation (right) with CD3/CD28 dynabeads. **(b)** Average percentage of positive cells (top panel: PD1 (p = 0.02), LAG3 (p = 0.06), TIGIT (p = 0.03), and TIM3 (ns)) and MFI (bottom panel: PD1 (p = 0.02), LAG3 (p = 0.03), TIGIT (p = 0.046), and TIM3 (ns)) of exhaustion markers on day 3 post-transduction with GFP or BATF3 OE (n = 3 individual donors, mean values +/− SEM, two-tailed paired t tests were used to determine statistical significance). **(c)** Time course of PD1, LAG3, TIGIT, and TIM3 expression post-transduction with GFP or BATF3 OE under acute or chronic stimulation (n = 3 individual donors, mean values +/− SEM).

In addition to LAG3, BATF3 silenced other canonical markers of T cell exhaustion including TIGIT, TIM3 and CISH (Fig. 3f and Extended Data Fig. 5c). We speculated these effects might be amplified in the context of chronic antigen stimulation (Extended Data Fig. 6). As previously observed^40^, PD1 expression peaked after the initial stimulation and then tapered off over time, whereas TIGIT, LAG3 and TIM3 expression was maintained or increased after each subsequent round of stimulation. Notably, BATF3 OE attenuated PD1 induction and restricted TIGIT, LAG3 and TIM3 expression to closely resemble that of acutely stimulated cells despite three additional rounds of TCR stimulation (Fig. 3g and Extended Data Fig. 6b,c). As terminally exhausted T cells often co-express multiple exhaustion-associated markers, we quantified the proportion of cells expressing each combination of TIGIT, LAG3 and TIM3. Only 13% of BATF3 OE T cells co-expressed all three markers compared to 65% and 59% of untreated and GFP-treated T cells (Fig. 3h).

### BATF3 OE remodels the epigenetic landscape

**Fig. 4:**
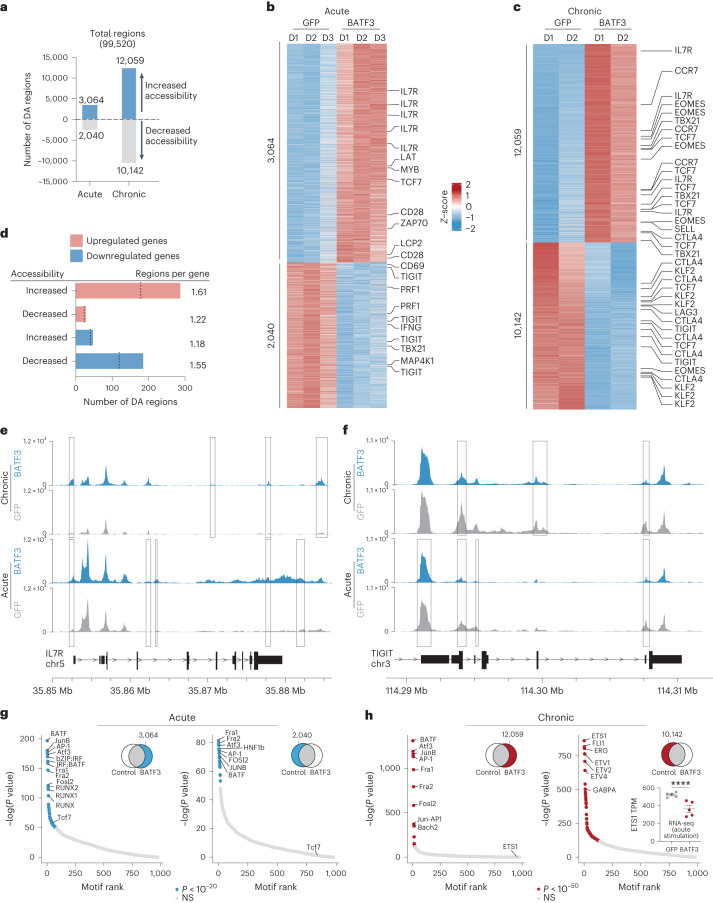
BATF3 OE remodels the chromatin landscape in the context of acute or chronic T cell stimulation. **a**, Number of ATAC-seq regions with increased or decreased accessibility in acutely (*n* = 3 donors) or chronically stimulated CD8^+^ T cells (*n* = 2 donors) with BATF3 OE on day 14 post-transduction. Differentially accessible (DA) regions were defined as *P*_adj_ < 0.05 using a paired two-tailed DESeq2 test with Benjamini–Hochberg correction. **b**,**c**, Heatmap of DA regions between control and BATF3 OE T cells under acute (**b**) or chronic (**c**) stimulation with selected regions annotated with their nearest gene. **d**, Joint analysis of RNA-seq and ATAC-seq datasets in the context of acute stimulation. Number of DA regions near upregulated and downregulated genes. Dashed lines represent the number of unique DEGs associated with DA regions. **e**,**f**, Representative ATAC-seq tracks of *IL7R* (**e**) and *TIGIT* (**f**) loci after acute or chronic stimulation with overlaid rectangles indicating DA regions between control and BATF3 OE T cells in each context. **g**,**h**, TF DNA-binding motifs enriched in open (left) and closed (right) regions of chromatin in BATF3 OE T cells compared to control T cells after acute (**g**) and chronic (**h**) stimulation. HOMER computes *P* values from the cumulative hypergeometric distribution and does not adjust for multiple hypotheses. Bar plot in lower right corner illustrates BATF3’s effect on *ETS1* expression based on RNA-seq (*n* = 5 donors, mean values ± s.e.m.; statistical significance was determined using a paired two-tailed DESeq2 test between treatment groups).

**Extended Data Fig. 7:**
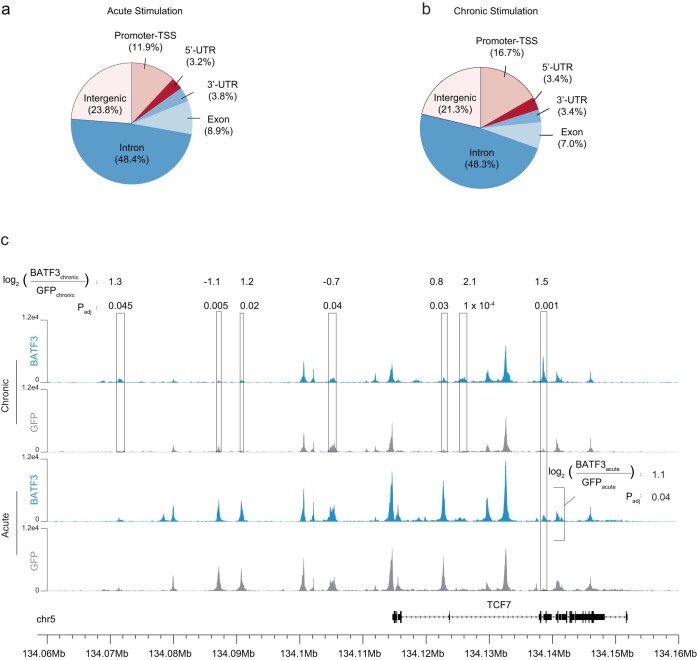
BATF3 OE remodels epigenetic landscape of TCF7 locus. Proportion of differentially accessible regions based on genomic feature classification with **(a)** acute stimulation and **(b)** chronic stimulation. **(c)** Representative ATAC-seq tracks of the TCF7 locus under acute and chronic stimulation with and without BATF3 OE.

As an orthogonal method of inducing T cell exhaustion, we acutely or chronically stimulated HER2-targeted CAR T cells with or without BATF3 OE with HER2^+^ cancer cells (Fig. 4, Supplementary Fig. 8 and Supplementary Note 8). We assessed chromatin remodeling by assay for transposase-accessible chromatin with sequencing (ATAC-seq) in response to BATF3 OE under acute or chronic stimulation. In both models, BATF3 OE extensively remodeled the chromatin with 5,104 and 22,201 differentially accessible regions compared to control T cells with 60% and 54% of these regions, respectively, being more accessible with BATF3 OE (Fig. 4a–c). Most of these changes were in intronic or intergenic regions consistent with *cis*-regulatory or enhancer elements (Extended Data Fig. 7a,b).

To understand whether changes in chromatin accessibility corresponded to changes in gene expression, we jointly analyzed our ATAC-seq and RNA-seq data in the context of acute stimulation. We assigned each differentially accessible region to its closest gene to estimate genes that could be regulated in *cis* by these elements. There was an enrichment of regions with increased or decreased accessibility proximal to upregulated and downregulated genes, respectively, indicating that BATF3-driven epigenetic changes affected nearby gene transcription (Fig. 4d). Approximately 25% of the genes that changed expression were associated with a corresponding differentially accessible region (297 out of 1,160 genes). For example, BATF3 OE increased accessibility at the *IL7R* promoter, intronic, 3′-untranslated region, and intergenic regions and decreased accessibility at the 5′-untranslated region, intronic and exonic regions of *TIGIT* (Fig. 4d,e). Additionally, BATF3 OE partially counteracted the effect of chronic antigen stimulation at each of these loci (Fig. 4d,e). Interestingly, BATF3 OE increased accessibility at regions near both memory (*TCF7*, *MYB*, *IL7R*, *CCR7* and *SELL*) and effector-associated genes (*EOMES* and *TBX21*) (Fig. 4c). This may represent a hybrid T cell phenotype or the presence of heterogenous subpopulations of memory and effector T cells. Consistent with RNA-seq and flow data, there was reduced accessibility at exhaustion-associated loci such as *TIGIT*, *CTLA4* and *LAG3* with BATF3 OE.

Next, we conducted motif enrichment analyses to gain further insight into the transcriptional networks regulating control and BATF3 OE T cells under acute and chronic stimulation (Fig. 4g,h). Compared to control T cells, AP-1 transcription family motifs were strongly enriched in both differentially open and closed regions with BATF3 OE under acute stimulation. In fact, 45% and 42% of differentially open and closed regions sites, respectively, harbored a BATF3 motif, suggesting direct BATF3 activity at these regions. This is consistent with the dual potential of BATF3 to silence or activate gene expression depending on its binding partners^41^. Interestingly, a TCF7 binding motif was uniquely enriched in differentially open regions with BATF3 OE. However, under chronic stimulation, AP-1 TF motifs were enriched with BATF3 OE only in differentially open regions. ETS family member motifs were enriched in closed regions, suggesting that BATF3 OE dampens the activity of these factors. Several ETS family members (for example *ETV1*, *ETV2* and *ETV4*) are not expressed at baseline in T cells, making it unlikely these genes contribute to the widespread epigenetic changes induced by chronic antigen stimulation. *ETS1*, however, may represent an important node of the transcriptional network as it is highly expressed at baseline (>500 transcripts per million, TPM) and significantly repressed by BATF3 OE under acute stimulation (Fig. 4h).

### BATF3 OE enhances potency of CAR T cells

**Fig. 5:**
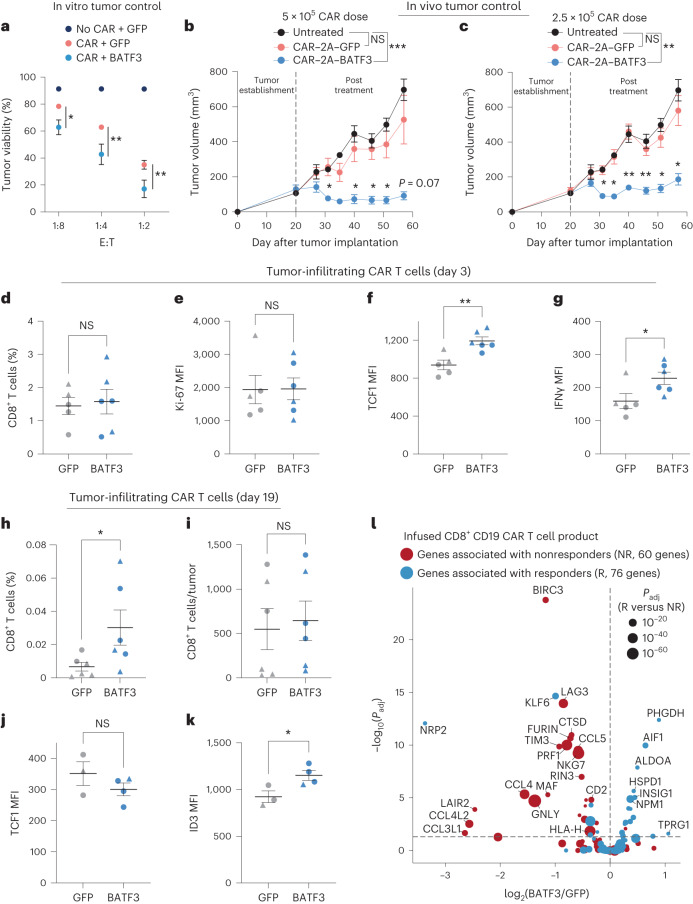
BATF3 OE enhances CAR T cell potency. **a**, Tumor viability after co-culture at specified E:T ratios (*n* = 3 donors). A two-way ANOVA with Dunnett’s post hoc test compared tumor viability at each E:T ratio: 1:8 (*P*_adj_ = 0.0243), 1:4 (*P*_adj_ = 0.0042) and 1:2 (*P*_adj_ = 0.0099). **b**,**c**, Tumor volumes of untreated (*n* = 5) and treated mice with 5 × 10^5^ (*n* = 1 donor, 5 mice per treatment) (**b**) or 2.5 × 10^5^ CAR T cells (*n* = 1 donor, 4 mice per treatment) (**c**) with or without BATF3 OE. Two-way ANOVA with Tukey’s post hoc tests compared tumor volumes at each time point across treatments. Tumor volumes were never different between untreated and control CAR groups. Asterisks indicate significant differences between control and BATF3 OE CAR T cells. **d**–**g**, Percentage of CD8^+^ T cells (**d**) within each resected tumor on day 3 post-treatment and (Ki-67 (**e**), TCF1 (**f**) and IFNγ (**g**) MFI of T cells (*n* = 2 donors, 2 GFP and 3 BATF3 mice for donor 1, 3 mice per treatment for donor 2). Two-tailed Mann–Whitney tests compared percentage of CD8^+^ cells and marker MFI between groups (*P* = 0.0065 for TCF1 and *P* = 0.0303 for IFNγ). **h**,**i**, Percentage (**h**) and total number (**i**) of CD8^+^ T cells within each resected tumor on day 19 post-treatment (*n* = 2 donors, 4 mice per treatment for donor 1, 2 GFP and 3 BATF3 mice for donor 2). Two-tailed Mann–Whitney tests compared percentage (*P* = 0.026) and total number of CD8^+^ cells between groups. **j**,**k**, TCF1 and ID3 MFI of T cells on day 19 (*n* = 2 donors, 1 mouse per treatment for donor 1, 2 GFP and 3 BATF3 mice for donor 2). Two-tailed *t*-tests compared MFI between groups (*P* = 0.037 for ID3). **l**, Significance (*P*_adj_) versus fold change between BATF3 OE and control CD8^+^ T cells for 144 genes associated with clinical outcome to CD19 CAR T cell therapy^38^. Mean values ± s.e.m. are plotted for **a**–**k**.

**Extended Data Fig. 8:**
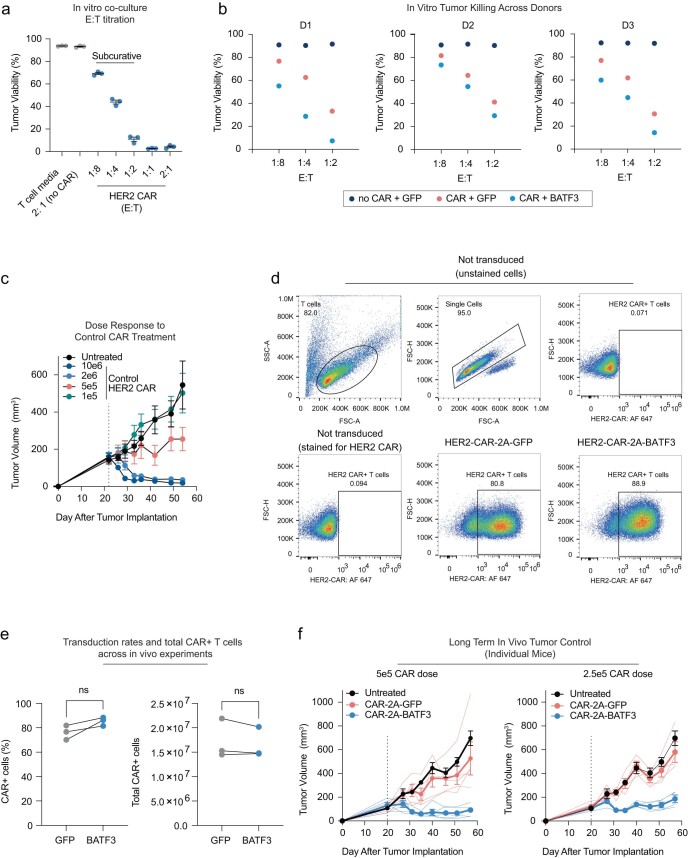
BATF3 OE enhances in vitro and in vivo tumor control. **(a)** Tumor viability after 24 hours of culture in T cell media, co-culture with CAR^null^ T cells, or co-culture with CAR T cells at specified effector to target (E:T) ratios (n = 3 donors, mean values +/− SEM). **(b)** Tumor viability after 24 hours of co-culture with GFP CAR^null^, GFP CAR + , and BATF3 OE CAR + CD8 T cells at specified E:T ratios for each donor. **(c)** Tumor volume over time as a function of the dose of control HER2 CAR T cells (n = 5 mice per treatment, mean values +/− SEM). Mice were intravenously injected with CAR T cells on day 21. **(d)** Representative flow plots of CAR expression in CD8+ T cells with control and BATF3 OE CAR lentiviral plasmids on day 9 post-transduction (the same day that the mice were intravenously injected with CAR T cells). **(e)** Summary statistics of transduction rates and total CAR+ T cells with control and BATF3 OE CAR lentiviral plasmids on day 9 post-transduction (n = 3 donors, lines connect donors across treatments, paired two-tailed t tests were used to determine statistical significance). **(f)** Tumor volumes of individual mice treated with 5 × 10^5^ (left panel, n = 5 mice per treatment group) or 2.5 × 10^5^ (right panel, n = 4 mice per treatment group) CAR T cells with or without BATF3 overexpression. Thinner lines represent tumor volumes of individual mice and thicker lines represent mean tumor volumes +/− SEM for each treatment group.

Given the profound transcriptional and epigenetic changes, we hypothesized that BATF3 OE might improve CD8^+^ T cell function. First, we observed that BATF3 OE increased killing of cultured human HER2^+^ cancer cells by HER2-targeted CAR T cells across donors and effector:target (E:T) ratios (Fig. 5a and Extended Data Fig. 8a,b). Next, we evaluated whether BATF3 OE could improve in vivo control of solid tumors, given the challenge of T cell exhaustion in the solid tumor setting^42,43^. To simplify delivery of the CAR and BATF3 transgenes, we constructed all-in-one lentiviral vectors encoding a HER2 CAR coupled to either GFP or BATF3 expression. Strikingly, CAR T cells co-expressing BATF3 markedly enhanced tumor control at two subcurative doses (2.5 × 10^5^ and 5 × 10^5^ CAR^+^ cells) compared to control CAR T cells in an orthotopic human HER2^+^ breast cancer model (Fig. 5b,c and Extended Data Fig. 8c–f).

**Extended Data Fig. 9:**
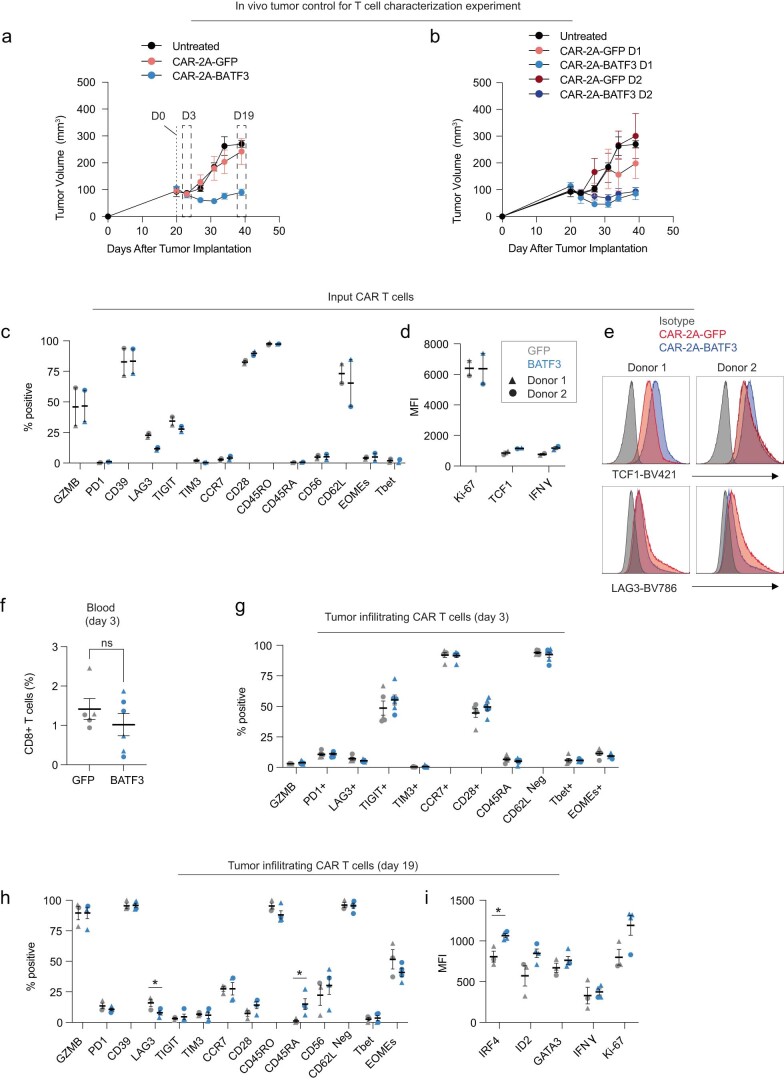
Characterization of CAR T cells with or without BATF3 OE during in vivo tumor control experiment. **(a)** Tumor volumes over time for untreated mice (n = 4 mice) and mice treated with 5 × 10^5^ CAR T cells with or without BATF3 overexpression (n = 2 donors, 4 GFP and 3 BATF3 mice for donor 1, 3 mice per treatment for donor 2. mean values +/− SEM). Input CAR T cells and tumor infiltrating CAR T cells on day 3 and day 19 post-treatment were characterized using flow cytometry. **(b)** Same as **(a)** except stratified based on donor (4 GFP and 3 BATF3 mice for donor 1, 3 mice per treatment for donor 2, mean values +/− SEM). **(c)** Percentage of positive cells or **(d)** MFI for indicated markers of input CAR T cells across groups (n = 2 donors, mean values +/− SEM). **(e)** Histograms of TCF1 and LAG3 expression for input CAR T cells. **(f)** Percentage of CD8+ T cells in peripheral blood on day 3 post-treatment across groups (n = 2 donors, 2 GFP and 3 BATF3 mice for donor 1 and 3 mice per treatment for donor 2, mean values +/− SEM). A two-tailed Mann-Whitney test was used to compare the percentage of CD8+ cells between the two groups. **(g)** Percentage of positive cells for indicated markers of tumor infiltrating CAR T cells on day 3 post-treatment across groups (n = 2 donors, 2 GFP and 3 BATF3 mice for donor 1 and 3 mice per treatment for donor 2, mean values +/− SEM). **(h)** Percentage of positive cells or **(I)** MFI for indicated markers of tumor infiltrating CAR T cells on day 19 post-treatment across groups (n = 2 donors, 1 mouse per treatment for donor 1, 2 GFP and 3 BATF3 mice for donor 2, mean values +/− SEM). Two-tailed t tests were used to compare expression of each marker between groups (% LAG3+ (p = 0.046) % CD45RA+ (p = 0.048), IRF4 MFI (p = 0.01)).

To explore the mechanism driving superior tumor control with BATF3 OE, we repeated the in vivo experiment with T cells from two different donors and phenotypically characterized the CAR T cells before treatment and after collecting tumor-infiltrating CAR T cells on day 3 and day 19 post-treatment (Fig. 5d–k, Extended Data Fig. 9 and Supplementary Fig. 9). Across both sets of experiments, there were no differences in CAR transduction rates (>70% for all groups) or the total number of CAR^+^ T cells before intravenous injections between CAR constructs (Extended Data Fig. 8d,e). Again, we observed superior tumor control with BATF3 OE CAR T cells across both donors (Extended Data Fig. 9a,b). Consistent with the previous characterization (Fig. 3f–h), input BATF3 OE cells tended to express lower levels of exhaustion markers including LAG3, TIGIT and TIM3 (Extended Data Fig. 9c).

More striking differences between the two groups emerged at the day 3 post-treatment time point. In control and BATF3 OE cells, we detected equivalent proportions of CD8^+^ T cells within the tumor and circulating in peripheral blood, indicating that BATF3 OE was not improving tumor control by merely increasing T cell proliferation or tumor trafficking (Fig. 5d and Extended Data Fig. 9f). Similarly, expression of the proliferative marker Ki-67 was equivalent between the groups (Fig. 5e). Rather, we noticed that tumor-infiltrating CAR T cells with BATF3 OE expressed higher levels of both TCF1 and IFN*γ* (Fig. 5f,g). This prompted us to revisit our gene expression and chromatin accessibility data. BATF3 OE did not increase expression of *TCF7* (which encodes for TCF1) under acute stimulation (Extended Data Fig. 5c). However, there were seven differentially accessible sites near the *TCF7* locus between control and BATF3 OE CAR T cells under chronic stimulation (Fig. 4c and Extended Data Fig. 7c). Notably, 5/7 sites were more accessible in BATF3 OE cells including all three intragenic regions. These data suggest that BATF3 OE can partially counter heterochromatinization of the *TCF7* locus during chronic antigen stimulation and retain higher levels of TCF1 expression.

As reflected in the tumor growth curves, we detected a higher proportion of tumor-infiltrating CAR T cells in the BATF3 OE group at the final day 19 time point, probably due to smaller tumor sizes, as the absolute number of T cells were similar between the two groups (Fig. 5h,i). We did not detect any CAR T cells in peripheral blood for either group. We stained the tumor-infiltrating CAR T cells for TCF1, TBET, EOMES, GATA3, ID2, ID3 and IRF4. Interestingly, TCF1 was no longer differentially expressed, but ID3 (a downstream TF of TCF1 (ref. ^44^)) was upregulated in the BATF3 OE group (Fig. 5j,k). Therefore, BATF3 OE T cells may have gradually transitioned from transcriptional programs driven by TCF1 to ID3.

Given the enhanced tumor control conferred by BATF3 OE in CD8^+^ T cells, we investigated whether BATF3 OE programmed a transcriptional signature associated with clinical response to ACT. In fact, nonresponders to CD19-targeted CAR T cell therapy had a significantly higher proportion of CD8^+^ T cells in a cytotoxic or exhausted phenotype compared to responders in a recent clinical trial^38^. Using these datasets, we identified 147 DEGs between the infused CD8^+^ CAR T cell product of responders and nonresponders (Supplementary Fig. 10). Of these 147 DEGs, 144 genes were detected in our RNA-seq data. Strikingly, BATF3 OE silenced 35% (23/65) of genes associated with nonresponse and activated 20% (16/79) of genes associated with response (Fig. 5l). Seven of the ten genes most strongly associated with clinical outcome were regulated in a favorable direction. Conversely, only 4.9% (7/144) of genes were regulated in a direction opposing positive clinical response, providing further evidence that BATF3 OE drives a transcriptional program associated with positive clinical outcomes.

### CRISPRko screens reveal cofactors of BATF3

**Fig. 6:**
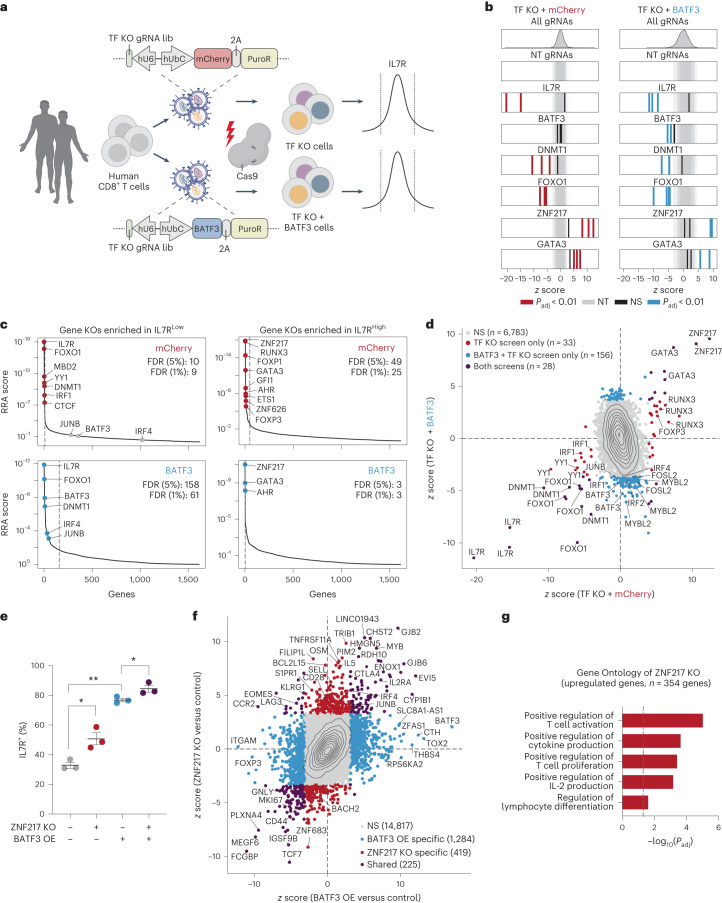
CRISPRko screens reveal cofactors of BATF3 and other targets for cancer immunotherapy. **a**, Schematic of CRISPRko screens with TF KO gRNA library (lib). **b**, *z* scores of gRNAs for selected genes in mCherry (left) and BATF3 (right) screens. Enriched gRNAs (*P*_adj_ < 0.01) were defined using a paired two-tailed DESeq2 test with Benjamini–Hochberg correction. **c**, Each gene target in the mCherry (top) and BATF3 (bottom) screens ranked based on the MAGeCK^58^ robust ranking aggregation (RRA) score in both IL7R^LOW^ (left) and IL7R^HIGH^ (right) populations. Dashed lines indicate FDR of 0.05. **d**, Scatter plot of *z* scores for each gRNA in CRISPRko screens with BATF3 versus without BATF3. Enriched gRNAs (*P*_adj_ < 0.01) were defined using a paired two-tailed DESeq2 test with Benjamini–Hochberg correction. **e**, Individual and combined effects of ZNF217 KO and BATF3 OE on IL7R expression (*n* = 3 donors, mean values ± s.e.m.). A one-way, paired ANOVA test with Tukey’s post hoc test was used to compare the percentage of IL7R^+^ cells between groups (*P*_adj_ = 0.041 for control versus ZNF217 KO, *P*_adj_ = 0.008 for control versus BATF3 OE, and *P*_adj_ = 0.049 for BATF3 OE versus BATF3 OE and ZNF217 KO). **f**, Scatter plot of transcriptomic effects of ZNF217 KO versus BATF3 OE relative to control T cells (*n* = 3 donors). DEGs (*P*_adj_ < 0.05) were defined using a paired two-tailed DESeq2 test with Benjamini–Hochberg correction and labeled on the basis of whether the DEG was unique to a specific perturbation or shared across perturbations. **g**, Selected enriched biological processes from ZNF217 KO. Statistical significance was defined using a two-tailed Fisher’s exact test followed by Benjamini–Hochberg correction.

BATF3 is a compact AP-1 TF with only a basic DNA binding domain and a leucine zipper motif. Given that BATF3 lacks additional protein domains such as transactivation domains for gene activation, we speculated that BATF3 interacts with other TFs to impact gene expression and chromatin accessibility (Supplementary Note 9)^41^. Additionally, we reasoned that other TFs might compete with or inhibit BATF3 and that removing these factors would further amplify the effects of BATF3 OE. To identify these factors, we conducted parallel CRISPRko screens with or without BATF3 OE using a gRNA library targeting all 1,612 human TF genes^45^ (TFome) (Fig. 6a). We selected IL7R expression as the readout for these screens because BATF3 OE profoundly increases IL7R expression (Fig. 3a,b), thus providing a proxy for BATF3 activity. IL7R is also expressed in 20–50% of CD8^+^ T cells at baseline, making it feasible to recover gene hits in both directions, unlike ubiquitously silenced and highly expressed genes.

**Extended Data Fig. 10:**
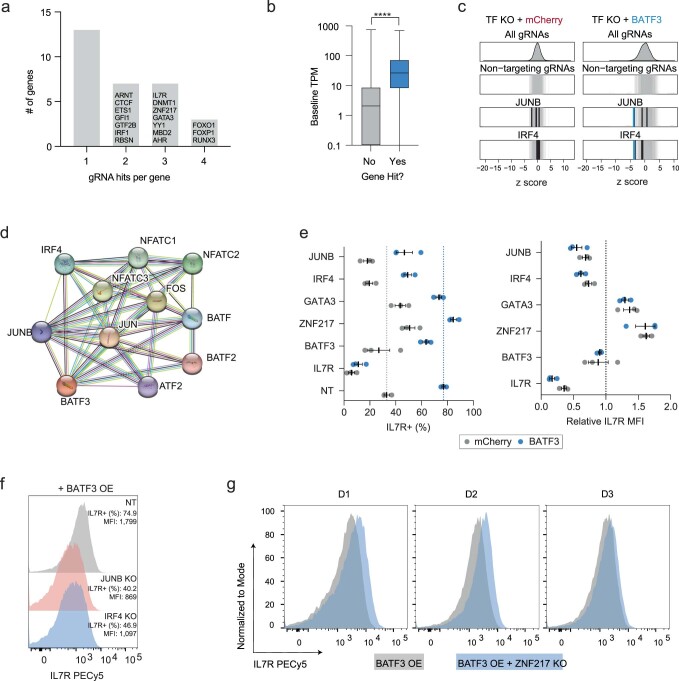
CRISPR knockout screens reveal co-factors of BATF3 and targets for cancer immunotherapy. **(a)** Number of gRNA hits (P_adj_ < 0.01 as defined by a paired two-tailed DESeq2 test with Benjamini-Hochberg correction) per gene in the CRISPRko screen without BATF3 OE. Only genes with at least 1 enriched gRNA were included in this plot. **(b)** Boxplot of baseline expression of genes stratified based on whether they were hits in the CRISPRko screen without BATF3 OE (n = 1,573 nonsignificant genes and n = significant 34 genes, genes with an FDR < 0.01 based on mageck gene-level analysis were classified as hits). Boxplots extend from the lower whisker (minimum value) to the upper whisker (maximum value). Lines represent the first quartile, median, and third quartile. A two-tailed t test was used to compare baseline expression of nonsignificant and significant gene hits. **(c)** z scores of gRNAs for JUNB and IRF4 in mCherry (left) and BATF3 (right) screens. Enriched gRNAs (P_adj_ < 0.01, labeled blue) were defined using a paired two-tailed DESeq2 test with Benjamini-Hochberg correction. Non-targeting gRNAs are labeled gray. **(d)** Predicted functional protein association network of BATF3 using STRING. **(e)** Percentage IL7R+ (left) and relative IL7R MFI (right) in CD8+ T cells with mCherry or BATF3 across gRNAs. Relative IL7R MFI was calculated by dividing the IL7R MFI of each targeting gRNA by the IL7R MFI of the non-targeting gRNA for each donor within the treatment group (n = 3 donors, mean values +/− SEM). **(f)** Representative histograms of IL7R expression in CD8+ T cells with BATF3 overexpression in combination with JUNB or IRF4 gene knockouts. **(g)** Effect of ZNF217 knockout on IL7R expression in CD8+ T cells across three donors with BATF3 OE.

As expected, *IL7R* gRNAs were the most enriched gRNAs in the IL7R low population across both screens (Fig. 6b). Notably, *BATF3* gRNAs only emerged in the screen with BATF3 OE as BATF3 is lowly expressed at baseline (Fig. 6b). *BATF3* gRNAs indiscriminately target endogenous and exogenous BATF3, indicating that knocking out exogenous BATF3 nullified its effects. Further supporting the robustness of these screens, we recovered multiple gRNA hits for many genes and the baseline expression of target gene hits was significantly higher than non-hit genes (Extended Data Fig. 10a,b).

By comparing gRNA- and gene-level enrichment between the two screens (Fig. 6c,d), we could classify whether genes regulated IL7R in a BATF3-independent or BATF3-dependent manner. For example, *FOXO1* and *DNMT1* were among the strongest hits in the IL7R low population for both screens, indicating BATF3-independent effects. To identify potential cofactors of BATF3, we searched for genes encoding for AP-1 or IRF TFs that were only enriched in the IL7R low population with BATF3 OE. Notably, *BATF3*, *JUNB*, and *IRF4* were the top genes meeting these criteria, confirming that BATF3 interacts with JUNB and IRF4 to mediate transcriptional control in CD8^+^ T cells (Fig. 6c and Extended Data Fig. 10c,d)^46^. These screens also revealed upstream regulators of IL7R and candidate gene targets for further improving ACT (Fig. 6c). The most enriched genes in the IL7R high population in the TF-knockout (KO) screen without BATF3 OE were *ZNF217*, *RUNX3*, *FOXP1*, *GATA3*, *GFI1*, *AHR*, *ETS1*, *ZNF626* and *FOXP3*. Fewer genes were enriched in IL7R high population in the BATF3 OE screen, in part because baseline IL7R expression was higher. Furthermore, we speculated that some TFs whose effects were lost with BATF3 OE might be downstream targets of BATF3. Indeed, the RNA-seq results show that several TFs including FOXP1, ETS1 and FOXP3 were all downregulated by BATF3 OE (Supplementary Table 4).

KO of three genes (*ZNF217*, *GATA3* and *AHR*) increased IL7R expression individually or in combination with BATF3 OE. *ZNF217* was the top hit in both screens and has not previously been characterized in the context of T cell biology. *GATA3* has been shown to promote CD8^+^ T cell dysfunction and targeted deletion of *GATA3* improves tumor control^47^. Moreover, both GATA3 and AHR can activate FOXP3 expression in regulatory T cells, providing further evidence of a link between T cell dysfunction and T cell regulatory activity^48–50^.

Next, we measured the effects of knocking out *IL7R*, *BATF3*, *JUNB*, *IRF4*, *ZNF217* and *GATA3* with and without BATF3 OE (Extended Data Fig. 10e). BATF3 OE alone increased IL7R expression by >40% compared to control CD8^+^ T cells (~33% to 77% IL7R^+^) (Extended Data Fig. 10e). Ablating BATF3 partially restored baseline IL7R levels, presumably due to incomplete nuclease activity across ectopic lentiviral copies of BATF3. IL7R induction by BATF3 was profoundly negated with either *JUNB* or *IRF4* KOs (Extended Data Fig. 10e,f). Conversely, *GATA3* and *ZNF217* KOs increased the percentage of IL7R^+^ T cells (Extended Data Fig. 10e). Finally, BATF3 OE and *ZNF217* KO together led to a further increase in T cells expressing IL7R (Fig. 6e and Extended Data Fig. 10g).

We next evaluated the transcriptional effects of ZNF217 or GATA3 KO relative to control T cells and BATF3 OE alone (Fig. 6f, Supplementary Fig. 11 and Supplementary Table 7). ZNF217 KO led to 644 DEGs relative to control T cells with many encoding for TFs and surface makers implicated in T cell biology and function (Fig. 6f). Further supporting a T cell-specific role for ZNF217, Gene Ontology analysis revealed that ZNF217 KO promoted positive regulation of T cell activation, proliferation, IL-2 production, and differentiation (Fig. 6g and Supplementary Table 7). Approximately 33% (225/644) of all DEGs with ZNF217 KO were shared with BATF3 OE with the vast majority (206/225) regulated in the same direction. Nevertheless, the majority of DEGs for each individual perturbation were unique, suggesting that ZNF217 KO and BATF3 OE can drive overlapping but also distinct transcriptional changes.

## Discussion

In this study, we developed an epigenetic screening platform with dSaCas9 to systematically map regulators of primary human CD8^+^ T cells through complementary CRISPRi/a screens. Our CRISPRi/a screens identified many regulators of CD8^+^ T cell with a striking convergence on BATF3. BATF3 OE markedly enhanced the potency of CD8^+^ CAR T cells in both in vitro and in vivo tumor models. The compact size of BATF3 makes it particularly amenable to integration into current ACT manufacturing processes by including it in the same lentivirus that delivers the CAR or TCR to donor T cells. It will be important to carefully assess the safety of ACT with T cells engineered with gene modules such as BATF3. Although the progeny of a single TET2^null^ CAR T cell clone cured a patient with advanced refractory chronic lymphocytic leukemia^20^, a recent study highlighted that biallelic deletion of *TET2* in combination with sustained expression of BATF3 can lead to antigen-independent clonal T cell expansion^51^. BATF3 OE alone does not induce adverse effects in T cells^52^, but the BATF–IRF axis can be oncogenic in the context of other genetic and epigenetic aberrations such as mutations, deletions, translocations and duplications^53–57^. We did not detect increased levels of *MYC* or *Ki-67* expression in our RNA-seq data nor did we detect elevated numbers of T cells after nearly 3 weeks of in vivo surveillance in tumor-bearing mice. Nevertheless, future work could focus on transiently delivering transgenes, modulating transgene expression or integrating suicide switches to control the activity of T cells in vivo.

The combination of TF OE with a TFome KO screen to dissect cofactors and downstream factors highlights the power of orthogonal CRISPR screen technologies. Specifically, these results support a model where BATF3 heterodimerizes with JUNB and interacts with IRF4 to drive transcriptional programs in CD8^+^ T cells. We also identified factors such as ZNF217 for further investigation, as these genes have not previously been associated with controlling T cell state or AP-1 gene regulation. Overall, this work expands the toolkit of epigenome editors and our understanding of regulators of CD8^+^ T cell state and function. This catalog of genes could serve as a basis for engineering the next generation of cancer immunotherapies.

## Methods

### Ethics statement

All animal experiments were conducted with strict adherence to the guidelines for the care and use of laboratory animals of the National Institutes of Health.

### Plasmids

All plasmids were cloned using Gibson assembly (NEB). The HER2 CAR constructs for in vivo tumor control studies were cloned by digesting an empty lentiviral vector (Addgene 79121) with MluI and amplifying HER2-CAR^59^ and 2A-GFP or 2A-BATF3 (gblock, IDT) fragments with appropriate overhangs for Gibson assembly. The following plasmids were deposited to Addgene: pLV hU6-gRNA hUbC-dSaCas9-KRAB-T2A-Thy1.1 (Addgene 194278) and pLV hU6-gRNA hUbC-VP64-dSaCas9-VP64-T2A-Thy1.1 (Addgene 194279).

### Cell lines

HEK293Ts and SKBR3s were maintained in Dulbecco’s modified Eagle medium (DMEM) GlutaMAX supplemented with 10% fetal bovine serum (FBS), 1 mM sodium pyruvate, 1× MEM non-essential amino acids, 10 mM HEPES, 100 U ml^−1^ penicillin and 100 μg ml^−1^ streptomycin. Jurkat lines were maintained in RPMI supplemented with 10% FBS, 100 U ml^−1^ penicillin and 100 μg ml^−1^ streptomycin. HCC1954s were maintained in DMEM/F12 supplemented with 10% FBS, 100 U ml^−1^ penicillin and 100 μg ml^−1^ streptomycin.

### Isolation and culture of primary human T cells

Human CD8^+^ T cells were obtained from either pooled peripheral blood mononuclear cell donors (ZenBio) using negative selection human CD8 isolation kits (StemCell Technologies) or directly from vials containing isolated CD8^+^ T cells from individual donors (StemCell Technologies). For technology development experiments, T cells were cultured in Advanced RPMI (Thermo Fisher) supplemented with 10% FBS, 100 U ml^−1^ penicillin and 100 μg ml^−1^ streptomycin. For T cell reprogramming experiments, T cells were cultured in PRIME-XV T cell Expansion XSFM (FujiFilm) supplemented with 5% human platelet lysate (Compass Biomed), 100 U ml^−1^ penicillin and 100 μg ml^−1^ streptomycin. All media were supplemented with 100 U ml^−1^ human IL-2 (Peprotech). T cells were activated with a 3:1 ratio of CD3/CD28 dynabeads to T cells and maintained at 1–2 × 10^6^ cells ml^−1^ unless otherwise indicated.

### Lentivirus generation and transduction of primary human T cells

For all technology development experiments, lentivirus was produced as previously described^60^. For all T cell reprogramming experiments, a recently optimized transfection protocol was used (Supplementary Method 1)^25^. Lentiviral supernatant was centrifuged at 600*g* for 10 min to remove cellular debris and concentrated to 50–100× the initial concentration using Lenti-X Concentrator (Takara Bio). T cells were transduced at 5–10% v/v of concentrated lentivirus at 24 h post-activation. For dual transduction experiments, T cells were serially transduced at 24 h and 48 h post activation.

### Design of *CD2*, *B2M* and *IL2RA* gRNA libraries

Saturation *CD2* and *B2M* CRISPRi gRNA libraries were designed to tile a 1,050-bp window (−400 bp to 650 bp) around the transcription start site (TSS) of each target gene using CRISPick^61^. The *IL2RA* CRISPRa gRNA library was designed to tile a 5,000-bp window (−4,000 bp to 1,000 bp) around the TSS of *IL2RA* using ChopChop^62^. Each gRNA library was designed to target dSaCas9’s relaxed protospacer adjacent motif (PAM) variant: 5′-NNGRRN-3′. NT gRNAs were generated for each library to match the nucleotide composition of the targeting gRNAs. *CD2*, *B2M* and *IL2RA* gRNA libraries are in Supplementary Table 1.

### gRNA library cloning

Oligonucleotide pools containing variable gRNA sequences and constant regions for polymerase chain reaction (PCR) amplification were synthesized by Twist Bioscience. gRNA amplicons were gel extracted, PCR purified and input into 20 μl Gibson reactions (5:1 molar ratio of insert to backbone) with 200 ng of Esp3I digested and 1 × solid-phase reversible immobilization (SPRI)-selected (Beckman Coulter) plasmid backbone. Gibson reactions were purified using ethanol precipitation and transformed into Lucigen’s Endura ElectroCompetent Cells. Transformed cells were cultured overnight and plasmids were isolated using Qiagen Midi Kits.

### CRISPRi tiling screens

CD8^+^ T cells from pooled peripheral blood mononuclear cell donors were transduced with all-in-one lentivirus encoding for dSaCas9–KRAB–2A–GFP and either CD2 (*n* = 2 replicates) or B2M (*n* = 3 replicates) gRNA libraries. Cells were expanded for 9 days and then stained for the target gene. Transduced cells in the lower and upper 10% tails of target gene expression were sorted for subsequent gRNA library construction and sequencing. All replicates were maintained and sorted at a minimum of 350× coverage.

### Construction of CRISPRa Jurkat lines and *IL2RA* CRISPRa tiling screens

Polyclonal dSaCas9^VP64^ and ^VP64^dSaCas9^VP64^ Jurkat cell lines were generated by transducing Jurkat cells with lentivirus encoding for either dSaCas9^VP64^–2A–PuroR or ^VP64^dSaCas9^VP64^–2A–PuroR. Cells were selected for 5 days using 0.5 µg ml^−1^ of puromycin. After selection, 1 × 10^6^ dSaCas9^VP64^ and ^VP64^dSaCas9^VP64^ Jurkat cells were plated and transduced in triplicate with the *IL2RA* gRNA library lentivirus at a low multiplicity of infection (MOI). Cells were expanded for 10 days, selected for Thy1.1 using a CD90.1 Positive Selection Kit (StemCell Technologies), and stained for Thy1.1 and IL2RA. Transduced cells in the lower and upper 10% tails of IL2RA expression were sorted for subsequent gRNA library construction and sequencing. All replicates were maintained and sorted at a minimum of 500× coverage.

### TF and epi-modifier CRISPRi/a gRNA library construction

Genes were selected on the basis of motif enrichment in differentially accessible chromatin across T cell subsets^4,63,64^ and a unified atlas of CD8 T cells in cancer and chronic infection^65^. *BACH2*, *TOX*, *TOX2*, *PRDM1*, *KLF2*, *BMI1*, *DNMT1*, *DNMT3A*, *DNMT3B*, *TET1* and *TET2* were manually added to the gene list (complete 121 member gene list is in Supplementary Table 2). The TSS for each gene was extracted using CRISPick and 1,000-bp windows were constructed around each TSS (−500 to +500 bp). After establishing an SaCas9 gRNA database with the strict PAM variant (NNGRRT) using guideScan^66^, the genomic windows were input into the guidescan_guidequery function to generate the gRNA library. Any gRNA that aligned to another genomic site with fewer than four mismatches was removed from the library. The final gRNA library contained at least seven gRNAs targeting 120/121 target gene (there were no *PBX2*-targeting gRNAs) with an average of 16 gRNAs per gene. A total of 120 NT gRNAs were included in the library for a total of 2,099 gRNAs (Supplementary Table 2).

### TF and epi-modifier CRISPRi/a gRNA screens

CD8^+^CCR7^+^ T cells were sorted and transduced with either CRISPRi (*n* = 2 donors) or CRISPRa (*n* = 3 donors) TF + epi-modifier gRNA libraries at a low MOI. Cells were expanded for 10 days and then stained for Thy1.1 (a marker to identify transduced cells) and CCR7 (a marker associated with T cell state). Transduced cells in the lower and upper 10% tails of CCR7 expression were sorted for subsequent gRNA library construction and sequencing. All replicates were maintained and sorted at a minimum of 300× coverage.

### Genomic DNA isolation, gRNA PCR and sequencing gRNA libraries

Genomic DNA was isolated using Qiagen’s DNeasy Blood and Tissue Kit. Genomic DNA was split across 100 μl PCR reactions (25 cycles at 98 °C for 10 s, 60 °C for 30 s, and 72 °C for 20 s) with Q5 2× Master Mix and up to 1 μg of genomic DNA per reaction. PCRs were pooled together for each sample and purified using double-sided (SPRI)bead selection at 0.6× and 1.8×. Libraries were run on a High Sensitivity D1000 tape (Agilent) to confirm amplicon size and quantified using Qubit’s dsDNA High Sensitivity assay. Libraries were diluted to 2 nM, pooled together at equal volumes, and sequenced using Illumina’s MiSeq Reagent Kit v2 (50 cycles). Primers are listed in Supplementary Table 5.

### Processing gRNA sequencing and gRNA analysis for FACS-based screens

FASTQ files were aligned to custom indexes for each gRNA library (generated from the bowtie2-build function) using Bowtie 2 (ref. ^67^). Counts for each gRNA were extracted and used for further analysis in R. Individual gRNA enrichment was determined using the DESeq2 (ref. ^68^) package to compare gRNA abundance between groups for each screen. DESeq2 results for promoter tiling screens, CRISPRi/a TF screens and CRISPRko screens are presented in Supplementary Tables 1, 2 and 7.

### Gene-level analysis for FACS-based TF CRISPRi and CRISPRa screens

DESeq2 *P* values were empirically transformed to cumulative probabilities using a midpoint linear interpolation of the 120 NT gRNA *P* values between 0 and 1. This transformation aligns the data with the null hypothesis that NT gRNA *P* values have a uniform distribution between 0 and 1. Within each gene, transformed *P* values were aggregated using a modified robust rank aggregation method to detect genes with nonuniform (non-null) gRNA *P* values. A gene-level *P* value was produced by comparison with 10 million gene-level null simulations of *P* values randomly sampled from a uniform distribution. NT gRNAs were randomly grouped into NT control ‘genes’ (NTCs) and analyzed in the same way. The number of gRNAs per NTC was sampled with replacement from the distribution of gRNAs per gene in the screen until all the NT gRNAs were used. Genes were selected as hits if their Benjamini–Hochberg false discovery rate (FDR) was less than 0.05. Gene-level aggregation was done in Python. Two effect sizes were computed for each gene by averaging gRNAs’ unshrunk DESeq2 log_2_FoldChange within the gene, weighted by each gRNA’s transformed one-sided *P* value. The larger (absolute value) effect size was chosen for each gene. Effect sizes were estimated in R. Gene-level effect sizes and *P* values are presented in Supplementary Table 2.

### gRNA validations

For *CD2* and *B2M* gRNA validations, CD8^+^ T cells were transduced in triplicate with each individual gRNA and followed the same timeline as the CRISPRi screens. For *IL2RA* gRNA validations, dSaCas9^VP64^ and ^VP64^dSaCas9^VP64^ Jurkat lines were transduced with each gRNA hit and followed the same timeline as the CRISPRa screen. Cells were stained with the respective antibody and measured using flow cytometry on day 9.

### Flow cytometry and surface marker staining

An SH800 FACS Cell Sorter (Sony Biotechnology) was used for cell sorting and analysis unless otherwise indicated. For antibody staining of all surface markers except CCR7, cells were collected, spun down at 300*g* for 5 min, resuspended in flow buffer (1× phosphate-buffered saline (PBS), 2 mM ethylenediaminetetraacetic acid and 0.5% bovine serum albumin) with the appropriate antibody dilutions and incubated for 30 min at 4 °C on a rocker. Antibody staining of CCR7 was carried out for 30 min at 37 °C. Cells were then washed with flow buffer, spun down at 300*g* for 5 min and resuspended in flow buffer for cell sorting or analysis. Antibody details are presented in Supplementary Table 5. Fluorescent minus one (FMO) controls were used to set appropriate gates for all flow panels.

### RT–qPCR

mRNA was isolated using Norgen’s Total RNA Purification Plus Kit. Reverse transcription was carried out by inputting an equal mass of mRNA for each sample into a 10 μl SuperScript Vilo cDNA Synthesis reaction. Two microliters of complementary DNA was used per PCR reaction with Perfecta SYBR Green Fastmix (Quanta BioSciences, 95072) using the CFX96 Real-Time PCR Detection System (Bio-Rad). All primers were designed using the National Center for Biotechnology Information’s primer blast tool, and amplicon products were verified by melt curve analysis. All RT–qPCRs are presented as log_2_ fold change in RNA normalized to *GAPDH* expression unless otherwise indicated. Primers are listed in Supplementary Table 5.

### scRNA-seq

A 40-gRNA library (Supplementary Table 3) containing all 32 gRNA hits from CRISPRi/a screens and 8 NT gRNAs was cloned into all-in-one CRISPRi and CRISPRa lentiviral plasmids. The experimental timeline for the scRNA-seq screens was identical to the cell sorting-based screens. CD8^+^CCR7^+^ T cells from three donors were transduced with CRISPRi and CRISPRa mini-TF gRNA libraries. T cells were expanded for 10 days and then stained and sorted for Thy1.1^+^ cells. Sorted cells were loaded into the Chromium X for a targeted recovery of 2 × 10^4^ cells per donor and treatment according to the Single Cell 5′-High-Throughput Reagent Kit v2 protocol (10x Genomics). SaCas9 gRNA sequences were captured by spiking in 2 μM of a custom primer into the reverse transcription master mix, as previously done for SpCas9 gRNA capture^36^. The custom primer was designed to bind to the constant region of SaCas9’s gRNA scaffold. 5′-Gene Expression (GEX) and gRNA libraries were separated using double-sided SPRI bead selection in the initial cDNA clean-up step. 5′-GEX libraries were constructed according to manufacturer’s protocol. gRNA libraries were constructed using two sequential PCRs (PCR 1: 10 cycles, PCR 2: 25 cycles). The PCR 1 product was purified using double-sided SPRI bead selection at 0.6 × and 2 ×. Twenty percent of the purified PCR 1 product was input into PCR 2. The PCR2 product was purified using double-sided SPRI bead selection at 0.6 × and 1 ×. All libraries were run on a High Sensitivity D1000 tape to measure the average amplicon size and quantified using Qubit’s dsDNA High Sensitivity assay. Libraries were individually diluted to 20 nM, pooled together at desired ratios and sequenced on an Illumina NovaSeq S4 Full Flow Cell (200 cycles) with the following read allocation: Read 1, 26; i7 index, 10; Read 2, 90. All oligos used in this study are listed in Supplementary Table 5.

### Processing and analyzing scRNA-seq

CellRanger v6.0.1 was used to process, demultiplex and generate UMI counts for each transcript and gRNA per cell barcode. UMI counts tables were extracted and used for subsequent analyses in R using the Seurat^69^ v4.1.0 package. Low-quality cells with <200 detected genes, >20% mitochondrial reads or <5% ribosomal reads were discarded. DoubletFinder^70^ was used to identify and remove predicted doublets. All remaining high-quality cells across donors for each treatment (CRISPRi or CRISPRa) were aggregated for further analyses. gRNAs were assigned to cells if they met the threshold (gRNA UMI >4). Cells were then grouped on the basis of gRNA identity. For differential gene expression analysis, we compared the transcriptomic profiles of cells sharing a gRNA to cells with only NT gRNAs using Seurat’s FindMarkers function to test for DEGs with the hurdle model implemented in model-based analysis of single-cell transcriptomics (MAST). All significant gRNA–gene links are listed in Supplementary Table 3. Upregulated DEGs were input into EnrichR’s GO Biological Process 2021 database^71^ for functional annotation.

### RNA sequencing

RNA was isolated using Norgen’s Total RNA Purification Plus Kit and submitted to Azenta (formerly Genewiz) for standard RNA-seq with polyA selection. Reads were first trimmed using Trimmomatic^72^ v0.32 to remove adapters and then aligned to GRCh38 using STAR v2.4.1a aligner. Gene counts were obtained with featureCounts^73^ from the subread package (version 1.4.6-p4) using the comprehensive gene annotation in Gencode v22. Differential expression analysis was determined with DESeq2 (ref. ^68^) where gene counts are fitted into a negative binomial generalized linear model and a Wald test determines significant DEGs. DESeq2 results of RNA-seq analyses with BATF3 OE and ZNF217 or GATA3 KO are presented in Supplementary Tables 4 and 7, respectively. Upregulated and downregulated DEGs were input into EnrichR’s GO Biological Processes 2021 database^71^ for functional annotation.

### scRNA-seq analysis of CD19 CAR T cell infusion product for responders and nonresponders

scRNA-seq data of the infused CD19 CAR T cell products from patients treated with tisagenlecleucel^38^ were downloaded from GEO: GSE197268. Patient data in MarketMatrix format were classified as responders (R) and nonresponders (NR) and processed with Seurat^74^ 4.2.0. For each patient, cells with fewer than 20% mitochondrial UMI counts, more than 20 GEX UMI counts, and in the bottom 95th percentile of GEX UMI counts were selected. GEX UMI counts were log-normalized for further analysis. Individual patient data were merged (merge function in Seurat) into a combined Seurat object, preserving the group identity in the cellular barcodes. GEX UMI counts were linearly scaled and centered (ScaleData function with default parameters) before finding the most DEGs (Seurat FindVariableFeatures) using principal component analysis. Clustering was performed using the first ten principal components to identify and select CD8^+^ T cells for subsequent analyses. MAST was used to identify DEGs between CD8^+^ T cells from responders and nonresponders. All DEGs between responders and nonresponders are presented in Supplementary Table 4.

### ATAC-seq

A total of 5 × 10^4^ transduced CD8^+^ T cells were sorted for Omni ATAC-seq as previously described^75^. Libraries were sequenced on an Illumina NextSeq 2000 with paired-end 50-bp reads. Read quality was assessed with FastQC and adapters were trimmed with Trimmomatic^72^. Trimmed reads were aligned to the Hg38 reference genome using Bowtie^76^ (v1.0.0) using parameters -v 2–best–strata -m 1. Reads mapping to the ENCODE hg38 blacklisted regions were removed using bedtools2 (ref. ^77^) intersect (v2.25.0). Duplicate reads were excluded using Picard MarkDuplicates (v1.130 (ref. ^78^)). Count-per-million-normalized bigWig files were generated for visualization using deeptools bamCoverage^79^ (v3.0.1). Peak calling was performed using MACS2 narrowPeak^80^ and filtered for *P*_adj_ ≤ 0.001. Peak calls were merged across samples to make a union-peak set. A count matrix containing the number of reads in peaks for each sample was generated using featureCounts^73^ (subread v1.4.6) and used for differential analysis in DESeq2 (ref. ^68^) (v.1.36). ChIPSeeker^81^ was used to annotate the genomic regions and retrieve the nearest gene around each peak. HOMER (v4.11) package^82^ was used to find transcription factor binding motifs that contributed to changes in chromatin accessibility with BATF3 OE compared to control cells (Supplementary Method 2).

### In vitro tumor killing assay

CD8^+^ T cells were transduced with lentiviruses encoding for a HER2–CAR–mCherry at 24 h post-activation and BATF3–2A–GFP or GFP at 48 h post-activation. After 12 days of expansion, CAR^+^GFP^+^ T cells were sorted and counted for the co-culture assay. Four hours before starting the co-culture, 2 × 10^5^ HER2^+^ SKBR3s were plated in a 24-well plate with cDMEM to allow the SKBR3s to adhere to the plate. After 4 h, cDMEM was discarded and mCherry^+^GFP^+^ T cells in cPRIME medium were added at the indicated E:T cell ratios. After 24 h of co-culture, the cells were collected by collecting the supernatant (containing T cells and dead tumor cells) and adherent cells (which were detached from the plate using trypsin). Cells were spun down at 600*g* for 5 min and then stained with a fixable viability dye and Annexin V to label dead and apoptotic cells according to manufacturer’s protocol (Supplementary Method 3).

### CD3/CD28 and tumor repeat stimulations

For chronic stimulation with CD3/CD28 dynabeads, cells were debeaded and counted, plated at 1–2.5 × 10^5^ T cells, and restimulated with fresh CD3/CD28 beads at a 3:1 bead-to-cell ratio in a 24-well plate every 3 days. On day 12, cells were stained and flow analyzed for expression of exhaustion-associated markers. For tumor restimulation, 1 × 10^5^ HER2 CAR T cells were transferred to a new 24-well plate with 2 × 10^5^ SKBR3s (1:2 E:T ratio) every 3 days. T cells were recovered without antigen stimulation for 2 days after the final round of tumor stimulation before ATAC-seq on day 14. In both assays, T cells were restimulated on days 3, 6 and 9.

### Mice

All experiments involving animals were conducted with strict adherence to the guidelines for the care and use of laboratory animals of the National Institutes of Health. All experiments were approved by the Institutional Animal Care and Use Committee at Duke University (protocol number A130-22-07). Six- to 8-week-old female immunodeficient NOD/SCID gamma (NSG) mice were obtained from Jackson Laboratory and then housed in 12-h light/dark cycles, at an ambient temperature (21 ± 3 °C) with relative humidity (50 ± 20%) and handled in pathogen-free conditions. Mice were euthanized before reaching a tumor volume of 2,000 mm^3^, the upper threshold defined by the Duke Institutional Animal Care and Use Committee.

### In vivo tumor model

A total of 2.5 × 10^6^ HER2^+^ HCC1954 cells were implanted orthotopically into the mammary fat pad of NSG mice in 100 μl 50:50 (v:v) PBS:Matrigel. T cells were expanded for 9–11 days post-transduction before treatment. Transduction rates were measured on the day of treatment using flow cytometry. For all in vivo experiments, transduction rates exceeded 70% for both HER2–CAR–2A–GFP and HER2–CAR–2A–BATF3 constructs. T cells were resuspended at 50 × 10^6^ CAR^+^ cells ml^−1^ in 1× PBS and serially diluted to the appropriate cell concentrations for 200-μl injections of either 10 × 10^6^, 2 × 10^6^, 5 × 10^5^, 2.5 × 10^5^ or 1 × 10^5^ HER2 CAR^+^ T cells. Then, 20–21 days after tumor implantation, and immediately before CAR T cell injections, mice were randomized into groups and tumors were measured. Tumor volumes were calculated on the basis of caliper measurements using the following formula: volume = ½(Length × Width^2^). CAR T cells were injected intravenously by tail vein. Tumors were measured every 4–6 days.

### Flow cytometry analysis of input and tumor-infiltrating CAR T cells

Mice bearing HCC1954 tumors were euthanized at days 3 and 19 post CAR T cell delivery under deep isoflurane anesthesia via exsanguination, from which blood was collected. Blood was processed via red blood cell lysis buffer (Sigma) treatment followed by washing in PBS. Tumors were resected, minced and incubated in RPMI-1640 medium (Gibco) for 45 min in 100 mg ml^−1^ Liberase (Sigma-Aldrich) and 10 mg ml^−1^ DNase I (Roche). Single-cell suspensions for blood and tumor were filtered through a 70-mm cell strainer (Olympus Plastics), washed in PBS (Gibco), stained with Zombie NIR (1:250, BioLegend), washed in FACS buffer (2% FBS (Sigma) + PBS), and treated with 1:50 mouse Tru-stain Fc block (BioLegend). Cells were then stained for cell surface markers followed by intracellular staining using the Transcription Factor Staining Buffer Set (Invitrogen) per the manufacturer’s instructions. Antibodies are listed in Supplementary Table 5, and more details on the staining protocol are outlined in Supplementary Method 4. All data were collected on a Fortessa X 20 (Duke Cancer Institute Flow Cytometry Core) and analyzed using Flow Jo V10.8.1. Blood/tumor from sham-infused mice and FMO controls were used to guide gating for CAR T cells and to confirm appropriate compensation, respectively.

### TFome CRISPRko gRNA library construction

The Brunello genome-wide KO^83^ library (four gRNAs per gene) was subset for 1,612 TFs^45^ and *IL7R*. A total of 550 NT gRNAs were included in the library for a total of 7,000 gRNAs (Supplementary Table 6). This gRNA library was cloned into SpCas9 gRNA lentiviral plasmids with either mCherry or BATF3.

### TFome CRISPRko screens and validations

A total of 20 × 10^6^ CD8^+^ T cells from two donors were activated with CD3/CD28 dynabeads at a 1:1 ratio. At 24 h post-activation, CD8^+^ T cells were split evenly and transduced in parallel with TFome CRISPRko gRNA libraries with mCherry or BATF3. At 48 h post-activation, cells were electroporated with Cas9 protein. Briefly, the cells were collected, spun down at 90*g* for 10 min, resuspended in 100 μl of Lonza P3 Primary Cell buffer with 3.2 μg Cas9 (Thermo) per 10^6^ cells, and electroporated with the pulse code EH115. After electroporation, warm medium was immediately added to each cuvette and cells were recovered at 37 °C for 20 min before being transferred into a six-well plate. On day 3 post transduction, cells were selected with 2 μg ml^−1^ of puromycin for 3 days. On day 9 post transduction, cells were stained for CD8, IL7R and a viability dye. Viable CD8^+^ T cells in the lower and upper 10% tails of IL7R expression were sorted for subsequent gRNA library construction and sequencing. All replicates were maintained and sorted at a minimum of 75× coverage. Subsequent individual gRNA validations were scaled down to 3.5 × 10^5^ cells per electroporation in an eight-well cuvette strip, but otherwise followed the same protocol and timeline as the CRISPRko screens.

### TFome CRISPRko screen analyses

gRNA enrichment was performed using DESeq2 as explained above. Gene-level enrichment was performed using the MAGeCK v.0.5.9.4 (ref. ^58^) test module with –paired and –control sgrna parameters, pairing samples by donors and NT gRNAs as control, respectively. Results from gRNA- and gene-level analyses are presented in Supplementary Table 6.

### Statistics and reproducibility

All statistical analysis methods are indicated in the figure legends (NS, not significant; *P < 0.05, **P < 0.01, ***P < 0.001, ****P < 0.0001). Statistical analyses and data visualizations were performed in Graphpad Prism v.9.0.2, R v4.2.1 or Python v3.7.6. All experiments have been replicated with at least two biological replicates. For in vivo studies, mice were randomly assigned into treatment groups. In this study, no statistical method was used to predetermine sample size, no data were excluded from the analyses, experiments were not randomized, and investigators were not blinded to allocation during experiments and outcome assessment.

### Reporting summary

Further information on research design is available in the Nature Portfolio Reporting Summary linked to this article.

## Online content

Any methods, additional references, Nature Portfolio reporting summaries, source data, extended data, supplementary information, acknowledgements, peer review information; details of author contributions and competing interests; and statements of data and code availability are available at 10.1038/s41588-023-01554-0.

### Supplementary information

Supplementary Information Supplementary Figs. 1–11, Notes 1–9 and Methods 1–4.

Reporting Summary

Supplementary Tables 1–7 Supplementary Table 1. *CD2*, *B2M*, *IL2RA* CRISPR tiling screening data. Supplementary Table 2. TF CRISPRi/a library and flow-based screening data. Supplementary Table 3. TF CRISPRi/a scRNA-seq data. Supplementary Table 4. BATF3 OE RNA-seq data. Supplementary Table 5. gRNAs, oligos and antibodies. Supplementary Table 6. CRISPR TFome KO screening data. Supplementary Table 7. ZNF217 and GATA3 KO RNA-seq data.

## Data Availability

All data associated with this study are present in the manuscript or its Supplementary Information files. GRCh38 reference genome was used for gRNA library designs and alignments. All CRISPR screening, scRNA-seq, RNA-seq and ATAC-seq data have been deposited in the Gene Expression Omnibus (GEO) under accession number GSE218988.

## Code availability

Publicly available software and packages were used in this study as indicated in Methods. A copy of the custom code used for gene-level analysis of the CRISPR screens is released on Zenodo^84^ (10.5281/zenodo.8370763).
