# Supplementary material for: Transcriptional and epigenetic regulators of human CD8+ T cell function identified through orthogonal CRISPR screens
Source: Nat Genet. 2023 Nov 9;55(12):2211–23. doi: 10.1038/s41588-023-01554-0 (PMC10703699; doi:10.1038/s41588-023-01554-0)
Supplement: Supplementary file 1 — Supplementary Figs. 1–11, Notes 1–9 and Methods 1–4. [file 41588_2023_1554_MOESM1_ESM.pdf]

nature genetics

Article

https://doi.org/10.1038/s41588-023-01554-0

Transcriptional and epigenetic regulators of human CD8$^+$ T cell function identified through orthogonal CRISPR screens

In the format provided by the authors and unedited

Table of Contents

Supplementary Figures

Supplementary Figure 1: Flow cytometry validation of CD2 and B2M gRNA hits and multiplex gene silencing 3

Supplementary Figure 2: Head-to-head comparison of ^{VP64}dSaCas9^{VP64} and ^{VP64}dSpCas9^{VP64} lentiviral titers and ^{VP64}dSaCas9^{VP64} activity in primary human CD8+ T cells 4

Supplementary Figure 3: TF and epigenetic modifier gRNA library design 5

Supplementary Figure 4: Representative gating and post sorts for initial and finals sorts for CRISPRi/a TF screens 6

Supplementary Figure 5: Effects of CRISPRi/a gRNA hits across donors 7

Supplementary Figure 6: Quality control of differential gene expression analyses for CRISPRi and CRISPRa TF scRNA-seq characterization 8

Supplementary Figure 7: Individual validation of subset of CRISPRi/a gRNAs on CCR7 expression 9

Supplementary Figure 8: Chronic antigen stimulation drives extensive chromatin remodeling in control CD8+ T cells 10

Supplementary Figure 9: Representative gating strategies for data in figures 5 and extended data 9 11

Supplementary Figure 10: Computational workflow for conducting differential gene expression analyses of CD19 CAR T cell products from responders and non-responders. 12

Supplementary Figure 11: Transcriptomic effects of GATA3 knockout 13

Supplementary Notes

Supplementary Note 1: Developing and characterizing dSaCas9 epigenetic screening platform 14-16

Supplementary Note 2: Design of TF gRNA library 17

Supplementary Note 3: Performing quality control of scRNA-seq data 18

Supplementary Note 4: Importance of gRNA coverage 19

Supplementary Note 5: MYB repression drives T cells towards an effector-like state 20
Supplementary Note 6: NR1D1 activation synthetically programs a transcriptional profile with features of T cell exhaustion 21
Supplementary Note 7: Exploring kinetics of BATF3 expression with CRISPRa and ORF 22
Supplementary Note 8: Chronic stimulation extensively remodels epigenetic landscape 23
Supplementary Note 9: Complex and highly specific transcription control mediated by AP-1 TFs 24

Supplementary Methods
Supplementary Methods 1: More detailed protocol of transfections for high-titer lentiviral production 25
Supplementary Methods 2: HOMER motif analyses 26
Supplementary Methods 3: Gating strategy for viable tumor cells in in vitro co-culture experiment 27
Supplementary Methods 4: Flow cytometry analysis of input and tumor infiltrating CAR T cells 28

References 29

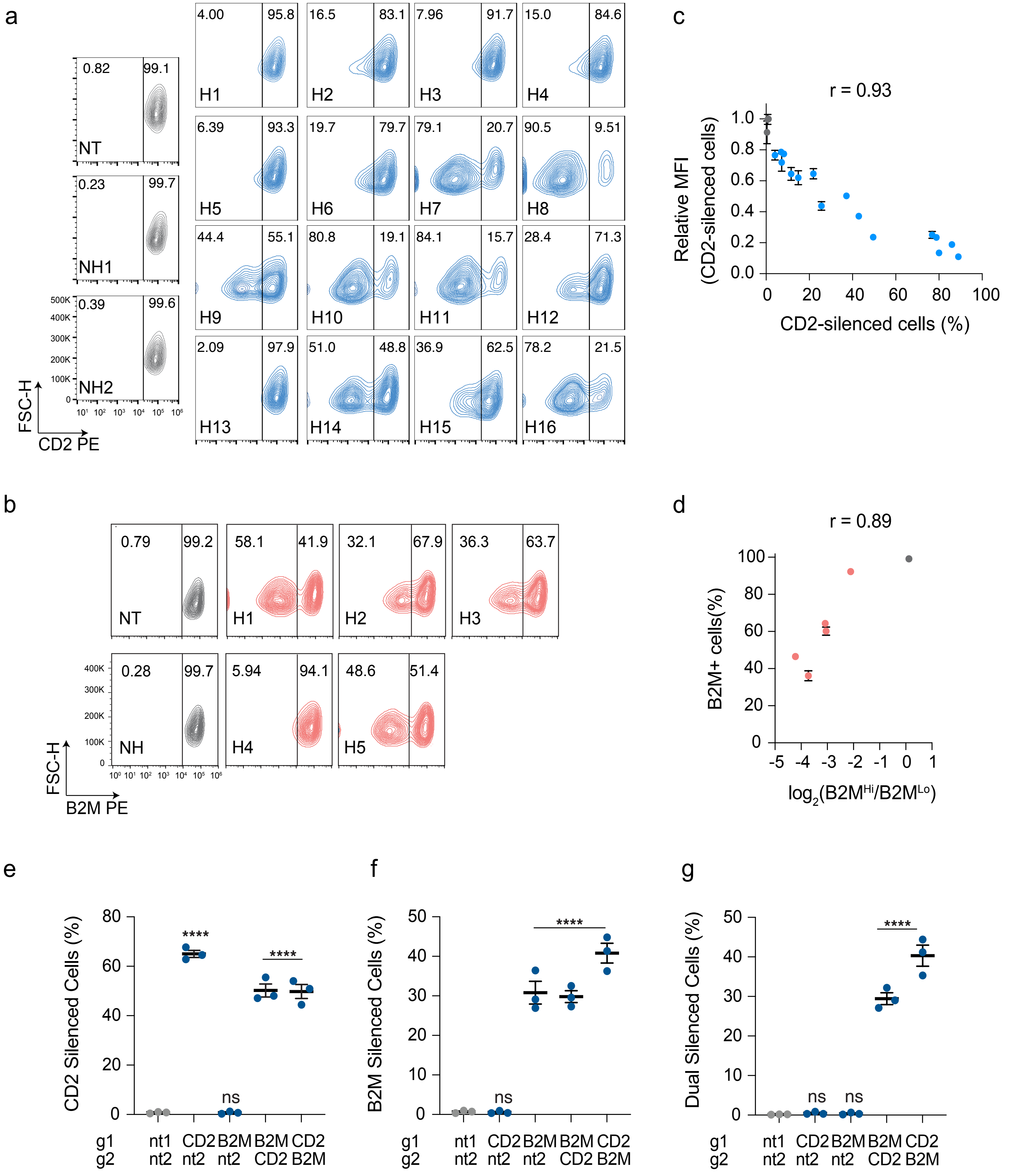

Supplementary Figure 1. Flow cytometry validation of CD2 and B2M gRNA hits and multiplex gene silencing. Representative contour plots of **(a)** CD2 and **(b)** B2M expression in CD8+ T cells across non-targeting (NT), non-hit (NH), and gRNA hits (H). **(c)** Relationship between relative MFI of CD2 silenced cells and the percentage of CD2 silenced cells (n = 18 CD2-targeting gRNAs (16 hits and 2 non-hits) and 1 non-targeting gRNA, mean values +/- SEM, Pearson's correlation coefficient (r) is indicated above the graph) **(d)** Relationship between B2M gRNA activity (n = 3 replicates of CD8+ T cells from pooled donors, mean values +/- SEM) and fold change enrichment in screen. Pearson's correlation coefficient (r) is indicated above the graph. Percentage of **(e)** CD2 silenced, **(f)** B2M silenced, and **(g)** dual CD2 and B2M silenced CD8+ T cells with the indicated pairs of non-targeting, CD2, and B2M gRNAs (n = 3 replicates of CD8+ T cells from pooled PBMC donors, mean values +/- SEM). One-way ANOVA with Tukey's post hoc tests were used to compare the percentage of CD2, B2M or dual CD2 and B2M silenced cells between gRNA combinations. g1 is driven by a human U6 promoter and g2 is driven by a mouse U6 promoter. CD2 H8 and B2M H1 gRNAs were used for multiplex gene silencing experiments.

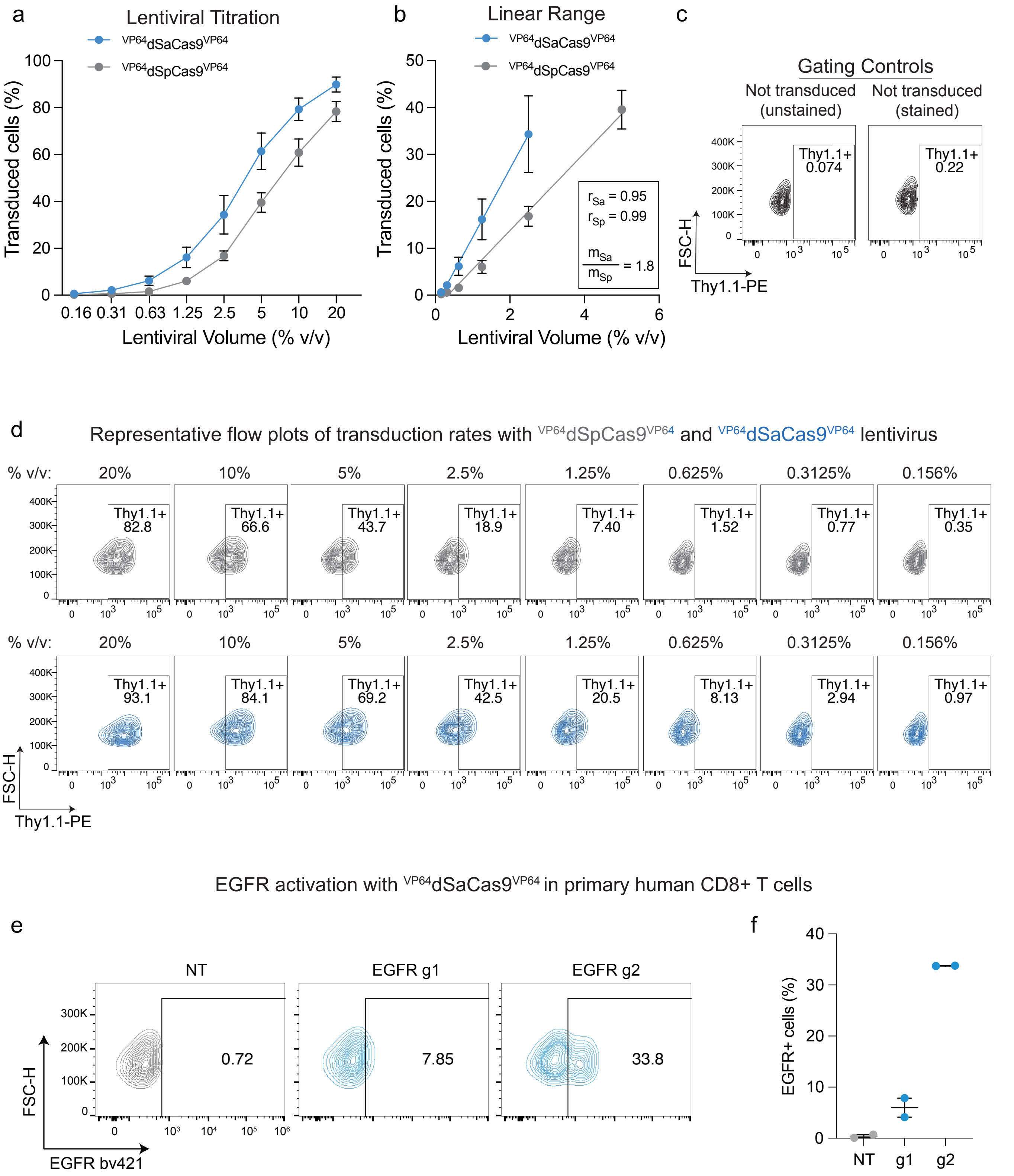

Supplementary Figure 2. Head-to-head comparison of ^{VP64}dSaCas9^{VP64} and ^{VP64}dSpCas9^{VP64} lentiviral titers and ^{VP64}dSaCas9^{VP64} activity in primary human CD8+ T cells.

(a) Transduction rate of primary human CD8+ T cells as a function of lentiviral volume for all-in-one ^{VP64}dSaCas9^{VP64} and ^{VP64}dSpCas9^{VP64} plasmids on day 9 post-transduction (n = 2 donors, mean values +/- SEM). **(b)** Linear range of transduction rate as a function of lentiviral volume. Pearson's correlation coefficient (r) and ratio of slopes were calculated using simple linear regression (n = 2 donors, mean values +/- SEM). **(c)** Flow cytometry controls used to set the Thy1.1+ gate for the lentiviral titer experiment. **(d)** Representative contour plots of Thy1.1 expression in CD8+ T cells transduced with serial titrations of ^{VP64}dSaCas9^{VP64} and ^{VP64}dSpCas9^{VP64} lentivirus. **(e)** Representative contour plots of EGFR expression in primary human CD8+ T cells on day 8 post-transduction with all-in-one lentiviruses encoding for ^{VP64}dSaCas9^{VP64} and either a non-targeting (NT) or an EGFR gRNA. **(f)** Summary statistics of EGFR activation (n = 2 replicates of CD8+ T cells from pooled PBMC donors, mean values +/- SEM).

a

TF + epi-modifier gRNA library	
Genes	120
Class	Curated TFs + epi-modifiers
Targets	Promoters
Average gRNAs per TSS	16
Non-targeting gRNAs	120
Total	2099

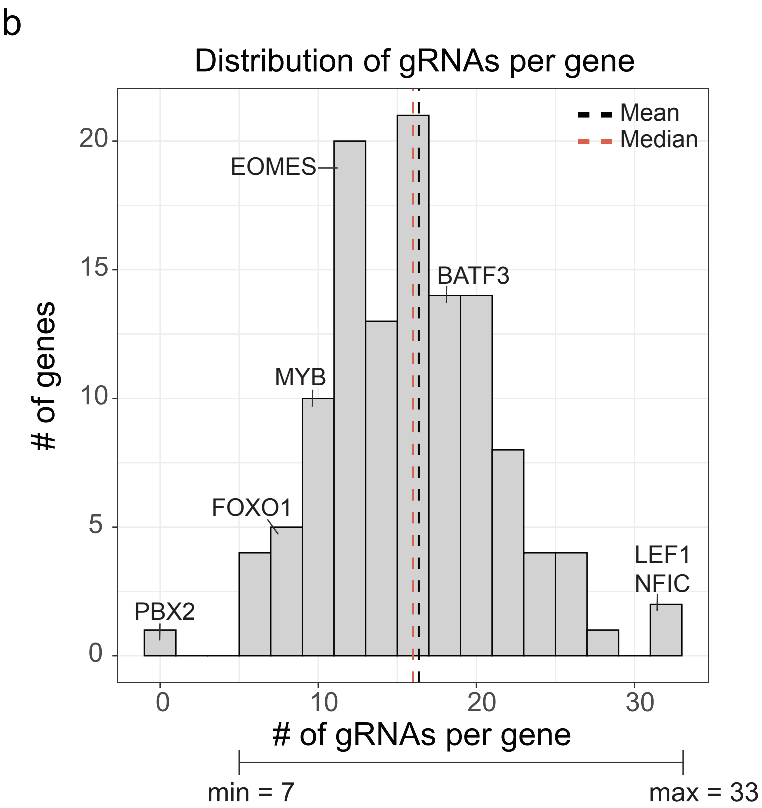

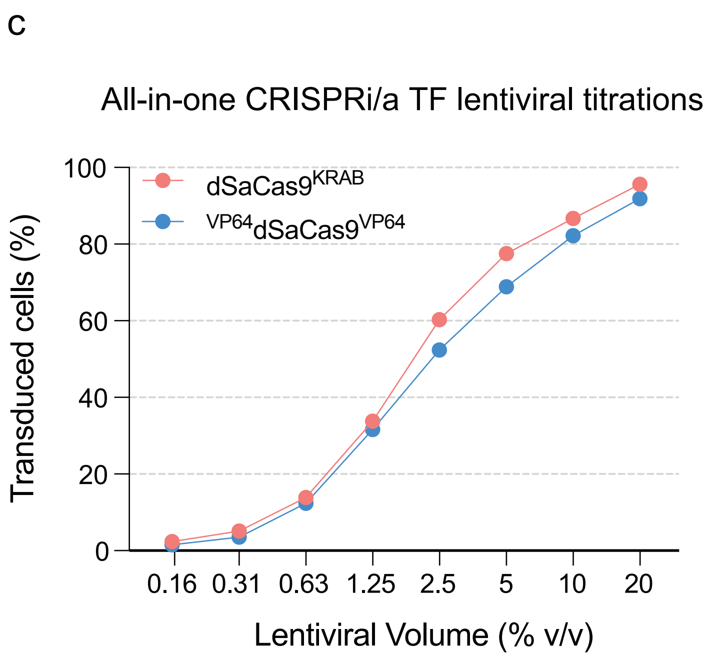

Supplementary Figure 3. TF and epigenetic modifier gRNA library design.
(a) Details of gRNA library targeting curated list of TFs and epigenetic modifiers **(b)** Histogram of gRNA representation across 121 candidate genes. **(c)** Transduction rate of primary human CD8+ T cells as a function of lentiviral volume for all-in-one CRISPRi and CRISPRa TF gRNA plasmids on day 9 post-transduction.

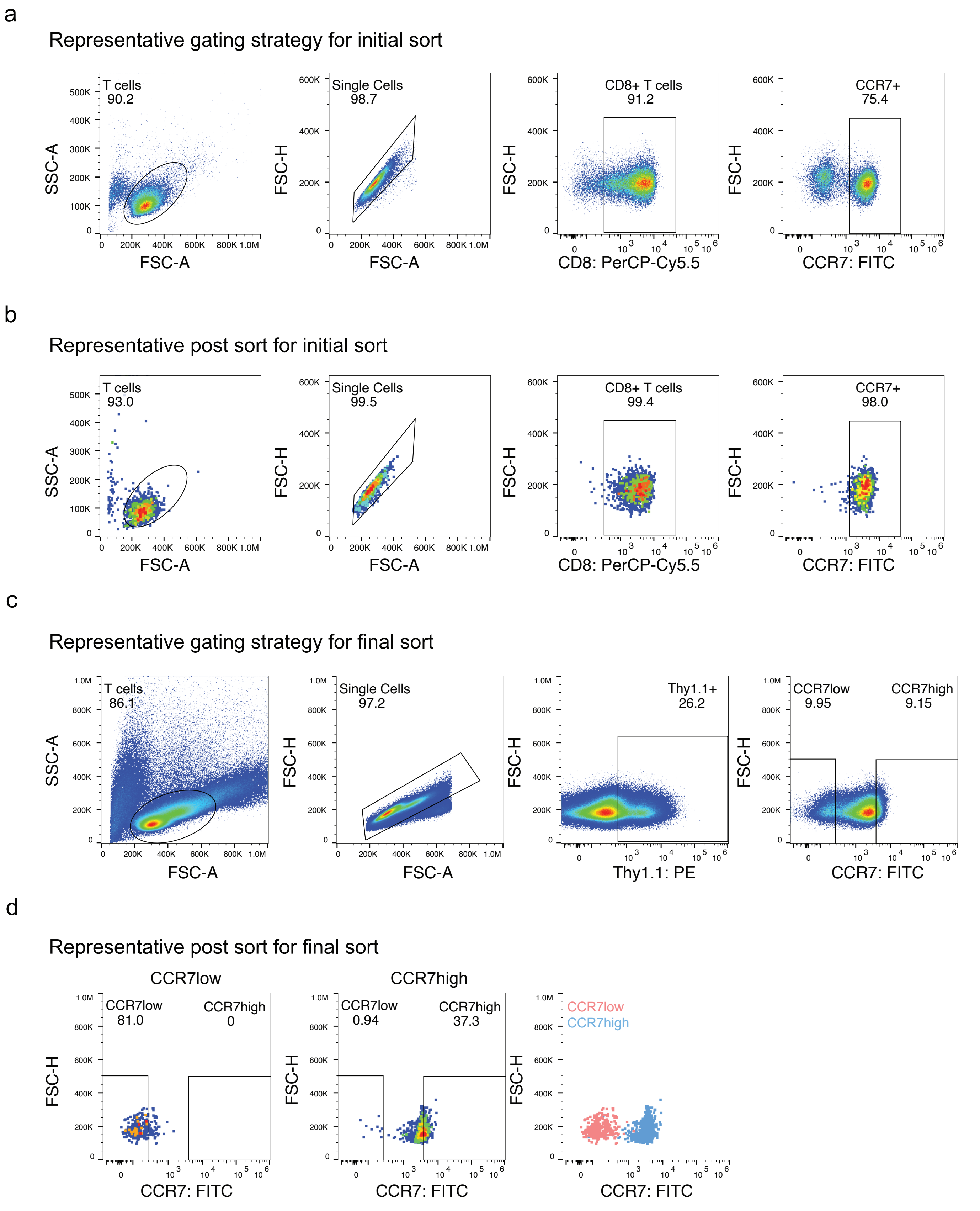

Supplementary Figure 4. Representative gating and post sorts for initial and final sorts for CRISPRi/a TF screens. **(a)** Representative initial gating for CD8+CCR7+ T cell population. **(b)** Representative post sort of CD8+CCR7+ T cell population. **(c)** Representative final gating strategy for transduced (Thy1.1+) cells in the lower and upper 10% tails of CCR7 expression on day 9 post-transduction. **(d)** Representative post sorts of Thy1.1+CCR7-low (left) and Thy1.1+CCR7-high (middle) populations. Overlay of sorted populations (right) shows clear separation of CCR7-low and CCR7-high populations.

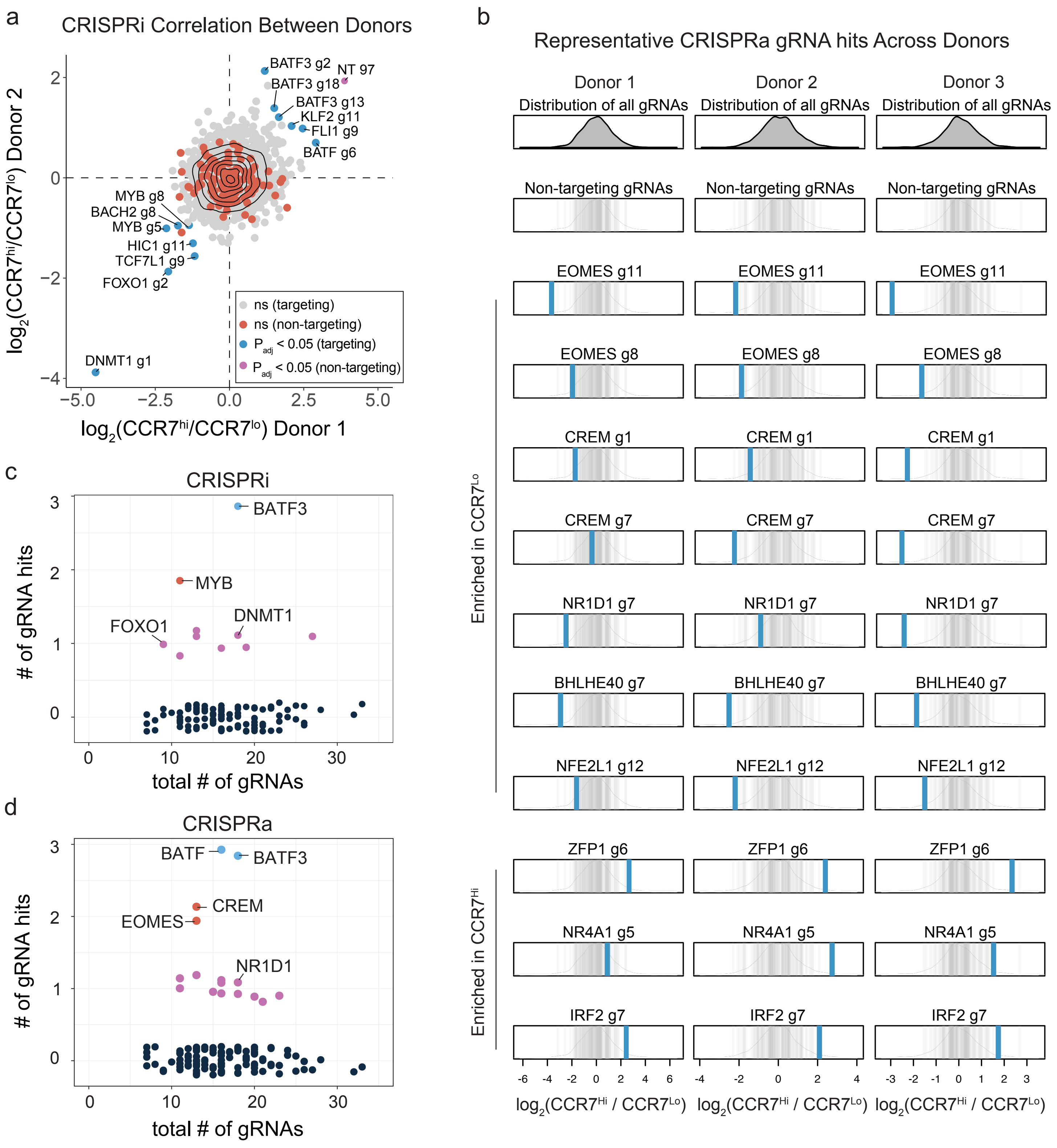

Supplementary Figure 5. Effects of CRISPRi/a gRNA hits across donors.
(a) Scatter plot of fold change in gRNA abundances between CCR7-high and CCR7-low populations across two donors in CRISPRi screens. P_{adj} values for each gRNA were defined using a paired two-tailed DESeq2 test with Benjamini-Hochberg correction. **(b)** Distribution of fold change in gRNA abundances between CCR7-high and CCR7-low populations for representative gRNA hits in the CRISPRa screen across three donors. Blue vertical lines represent gRNA hits and gray vertical lines represent the distribution of 120 non-targeting gRNAs. Scatter plots of number of gRNA hits versus total number of gRNAs for each gene in **(c)** CRISPRi and **(d)** CRISPRa TF screens. A slight vertical stagger was implemented for visualization purposes, but there are only discrete values as denoted by the colors (0 gRNA hits = black, 1 gRNA hit = magenta, 2 gRNA hits = red, 3 gRNA hits = blue).

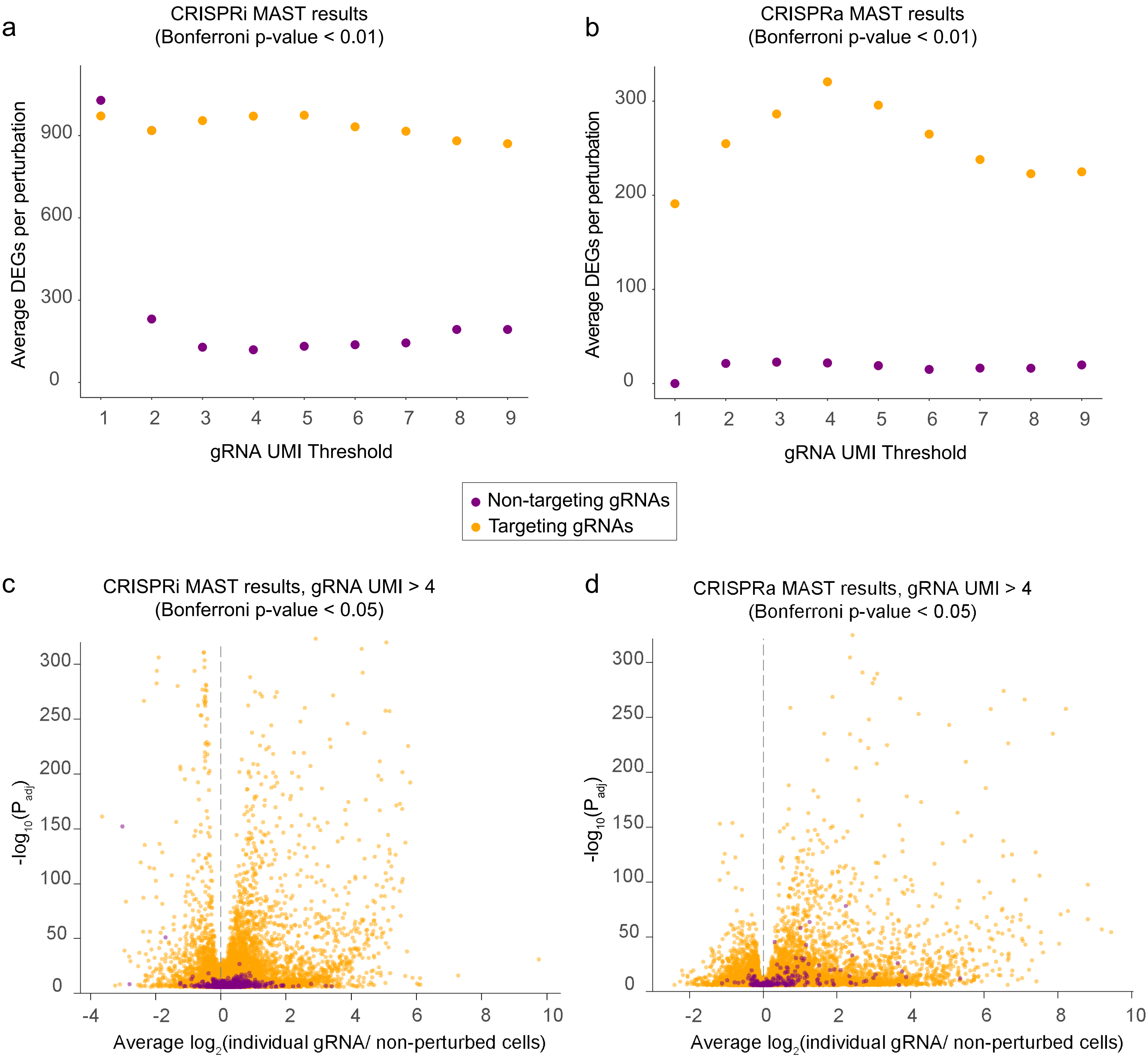

Supplementary Figure 6. Quality control of differential gene expression analyses for CRISPRi and CRISPRa TF scRNA-seq characterization. The average number of differentially expressed genes (DEGs) for targeting and non-targeting gRNAs as a function of gRNA UMI threshold used for gRNA assignment to cells in **(a)** CRISPRi and **(b)** CRISPRa scRNA-seq screens. Volcano plots of **(c)** CRISPRi and **(d)** CRISPRa scRNA-seq screens with the statistical significance (P_{adj}) of each significant gRNA-gene pair plotted versus the fold change in gene expression relative to non-perturbed cells. P_{adj} values for each gRNA-to-gene link in this figure were defined using MAST's two-tailed test with Bonferroni correction.

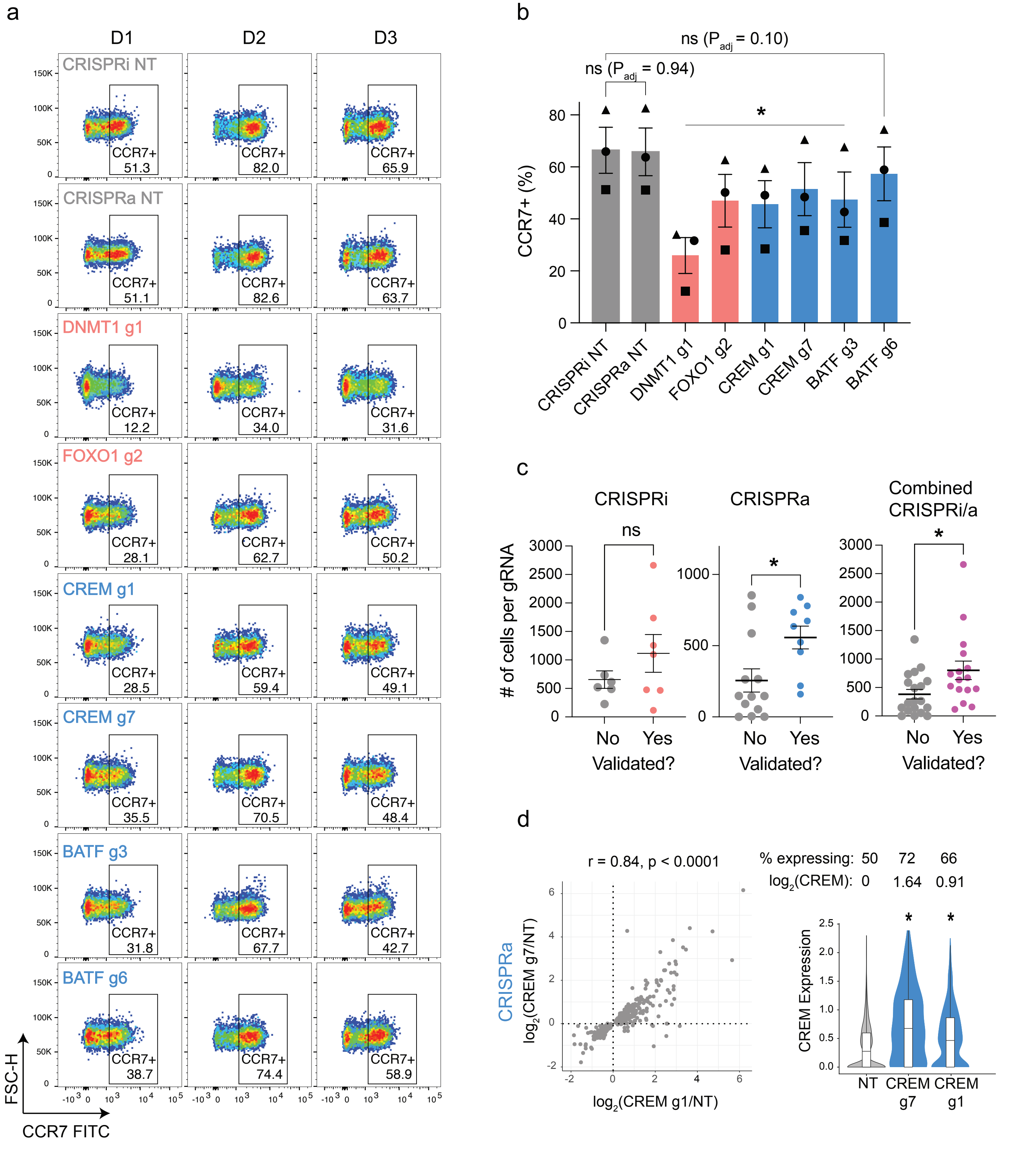

Supplementary Figure 7. Individual validation of subset of CRISPRi/a gRNAs on CCR7 expression.
(a) Flow cytometry plots of CCR7 expression in CD8+ T cells across three donors with the indicated CRISPRi/a perturbations. **(b)** Summary statistics of percentage CCR7+ T cells across CRISPRi/a perturbations. A paired one-way ANOVA test was used to compare the percentage of CCR7+ T cells for each perturbation to the CRISPRi NT treatment group (n = 3 donors, mean values +/- SEM, different shapes are used to denote each donor). **(c)** Number of cells assigned to each gRNA across CRISPRi (left, n = 6 unvalidated gRNAs and n = 7 validated gRNAs), CRISPRa (middle, n = 13 unvalidated gRNAs and n = 9 validated gRNAs, p = 0.0208), and joint CRISPRi/a (right, n = 19 unvalidated gRNAs and n = 16 validated, p = 0.0224) scRNA-seq datasets with gRNAs stratified based on whether they affected CCR7 expression as predicted by the flow-based screen. A two-tailed Mann-Whitney test was used to determine statistical significance for each group (mean values +/- SEM) **(d)** Correlation of the union set of DEGs between CREM gRNAs from CRISPRa scRNA-seq characterization (left, n = 474 genes, Pearson's correlation coefficient followed by a two-tailed t test). Violin plots of CREM expression across cells assigned to indicated gRNA with the fold change in target gene expression relative to NT and the percent of cells expressing the target gene indicated above (right, n = 2,980 cells with NT, n = 142 cells with CREM g7 (P_{adj} = 1.0e-39) , n = 635 cells with CREM g1 (P_{adj} = 6.8e-31), P_{adj} values were defined using a two-tailed MAST test with Bonferroni correction). Boxplots extend from the lower whisker (smallest value within 1.5 IQR of the first quartile) to the upper whisker (largest value within 1.5 IQR of the third quartile). The boxed lines represent the first quartile, median, and third quartile.

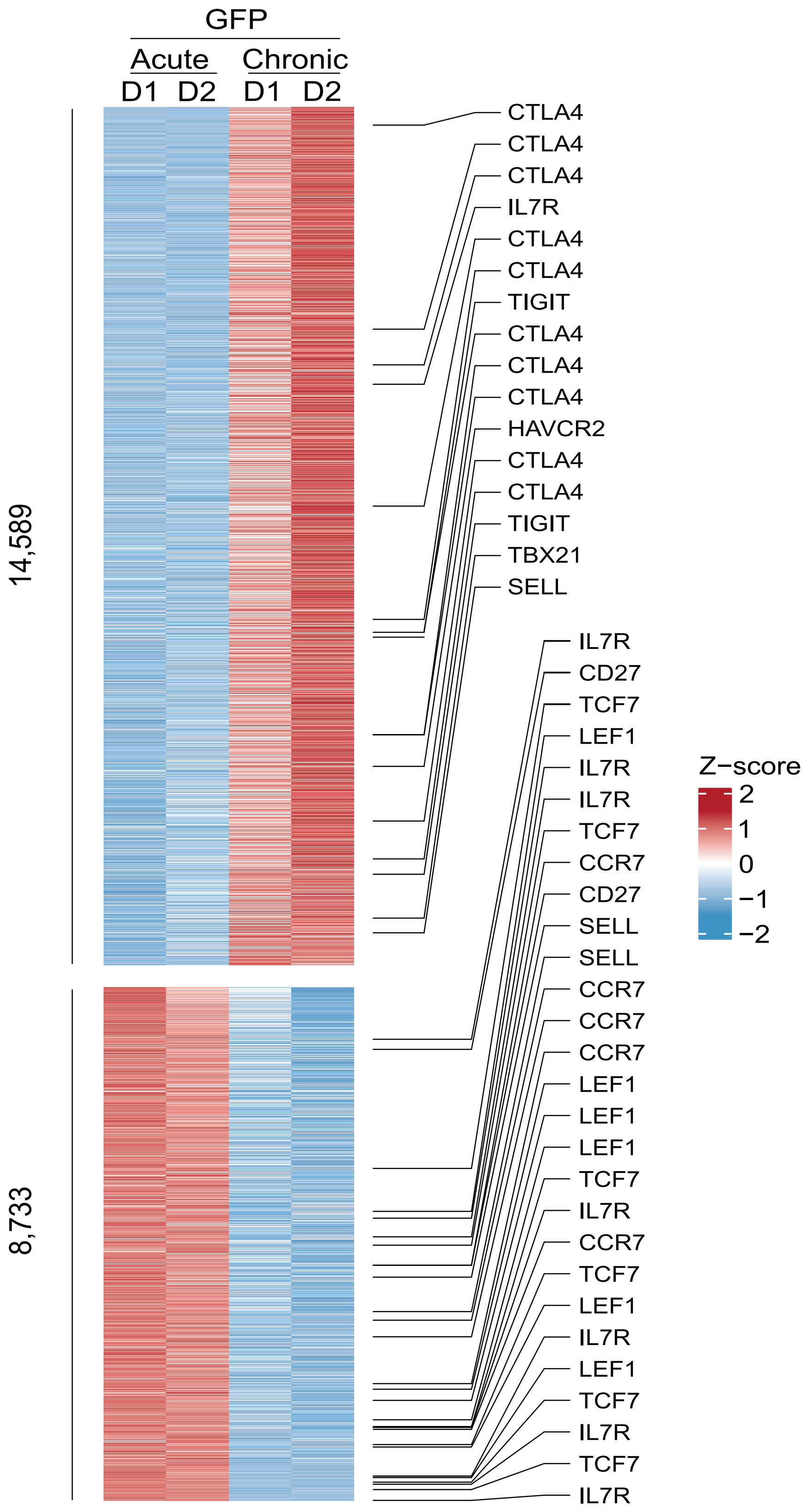

Supplementary Figure 8. Chronic antigen stimulation drives extensive chromatin remodeling in control CD8+ T cells.
Heatmap of differentially accessible regions with selected regions annotated with their nearest gene.

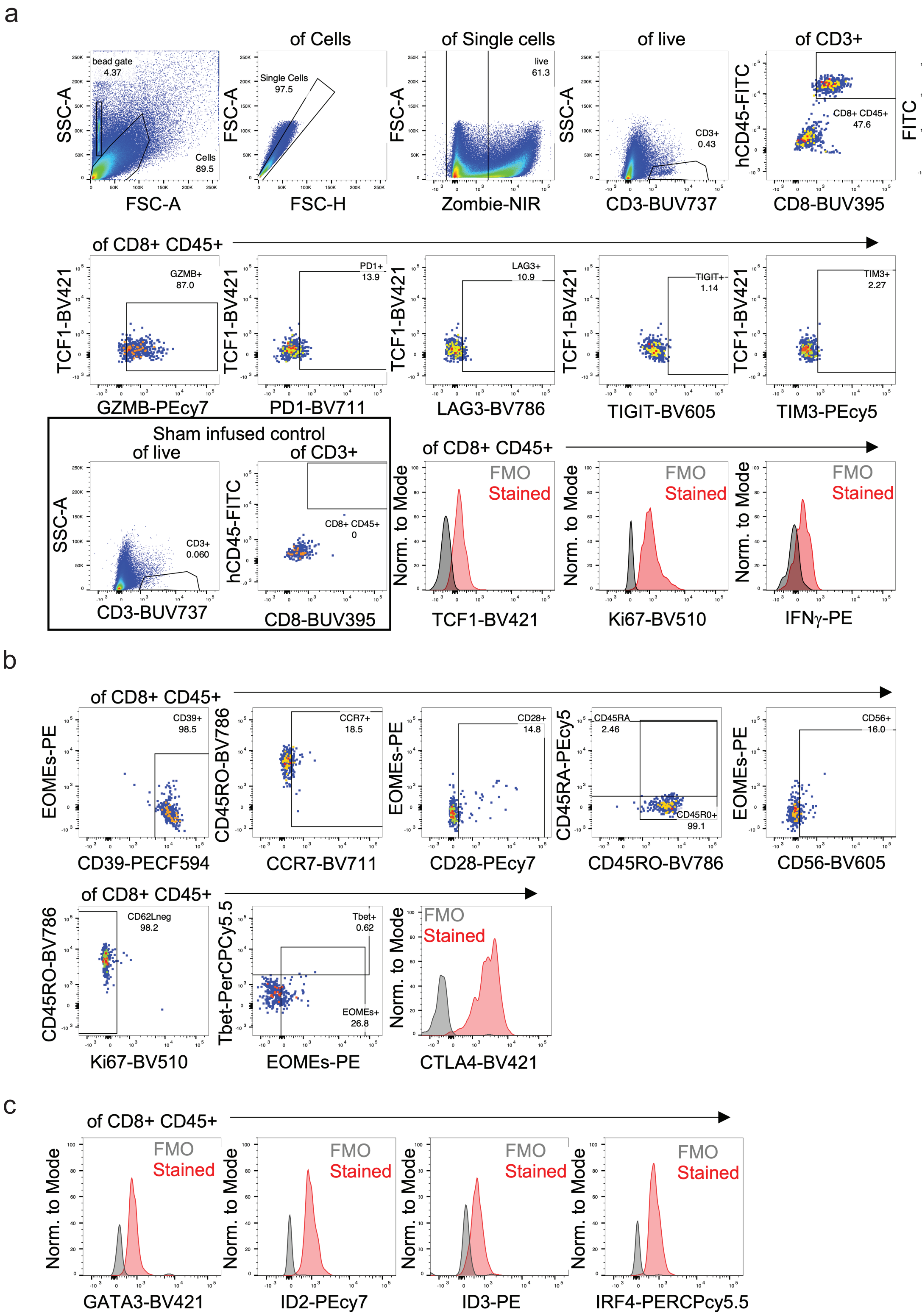

Supplementary Figure 9. Representative gating strategies for data in figures 5 and extended data 9. **(a-c)** Dissociated single cell suspensions from tumors at day 19 post treatment were gated for live singlets, followed by identification of CD3+, CD8+, and human (h)CD45+ to identify CAR T cells as shown. Identified T cells were then stained with three panels [see methods and supplementary table 5: panels 1 (A), 2 (B) and 3 (C) for indicated targets]. Fluorescence minus one (FMO) controls were used to confirm appropriate compensation and determine positivity for each marker, relevant markers are shown in histograms comparing FMO vs antibody staining.

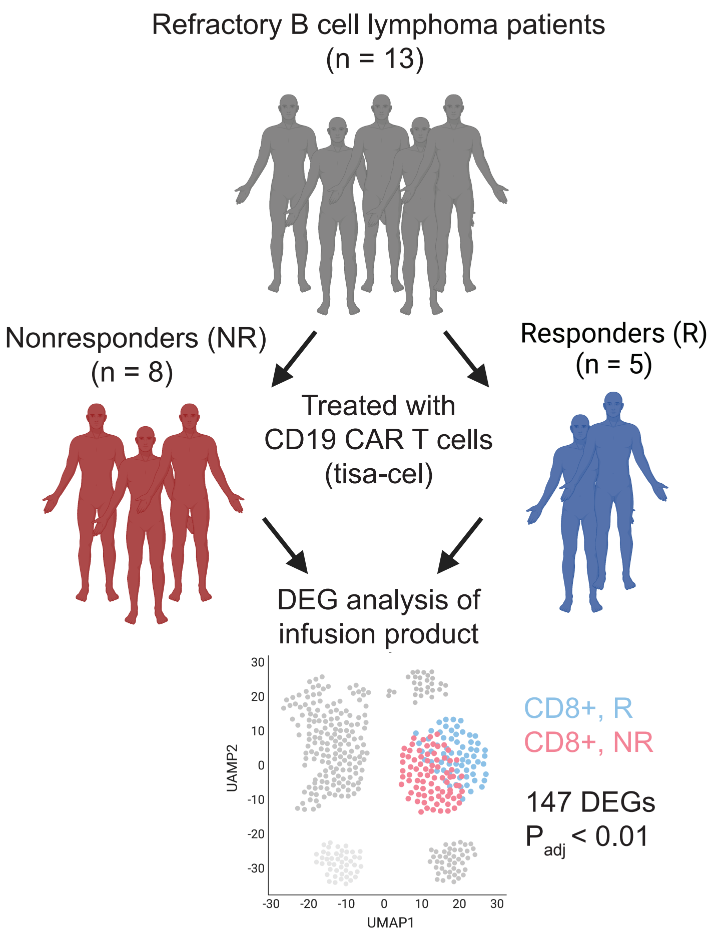

Supplementary Figure 10. Computational workflow for conducting differential gene expression analyses of CD19 CAR T cell products from responders and non-responders.

Patients were stratified based on clinical response to CD19 CAR T cell therapy and differential gene expression analysis was then performed on CD8+ T cells between non-responders and responders.

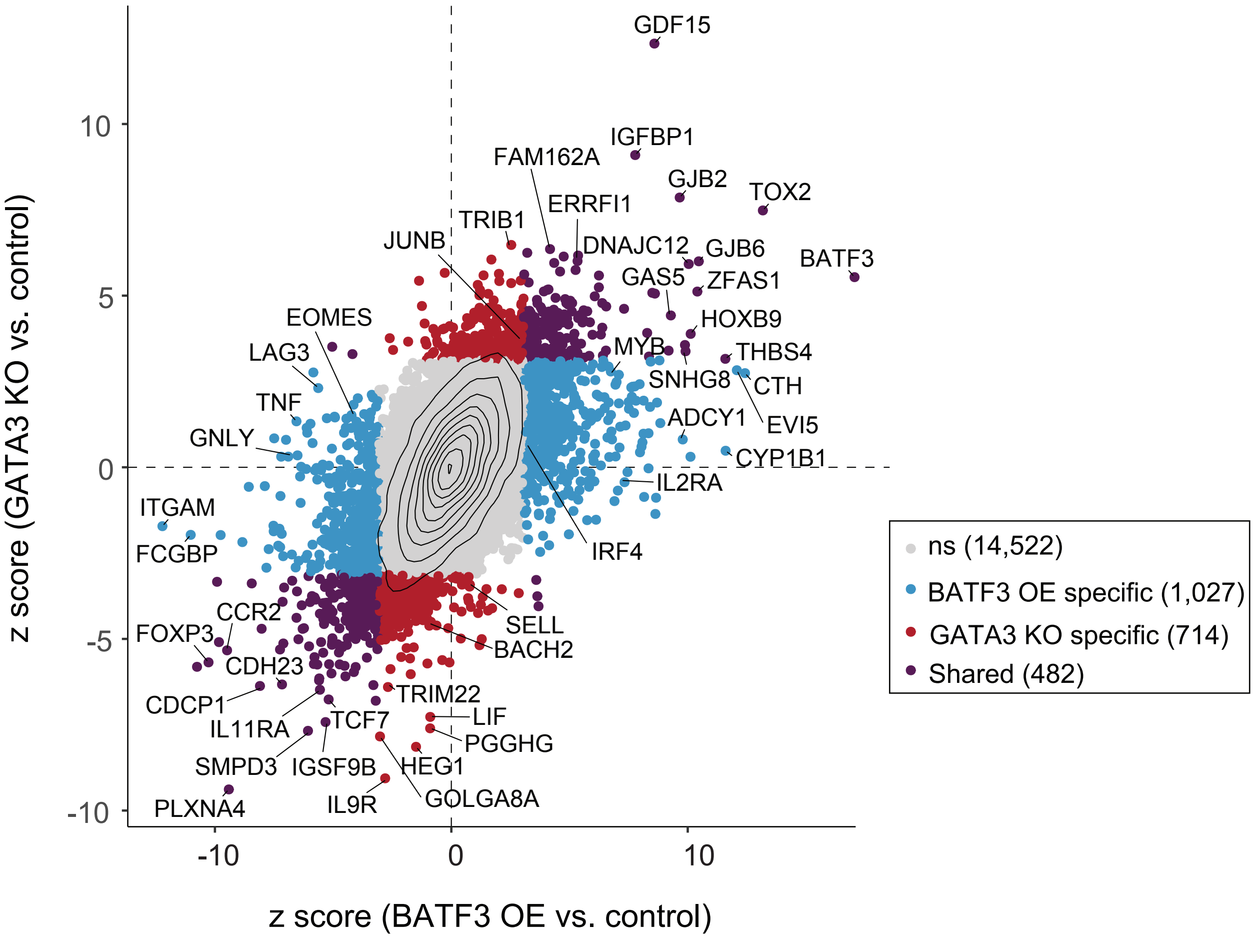

Supplementary Figure 11. Transcriptomic effects of GATA3 knockout.
Scatter plot of transcriptomic effects of GATA3 knockout versus BATF3 OE relative to control T cells. DEGs ($P_{adj} < 0.05$) were defined using a paired two-tailed DESeq2 test with Benjamini-Hochberg correction and labeled based on whether the DEG was unique to a specific perturbation or shared across perturbations.

Supplementary notes

Supplementary Note 1: Developing and characterizing dSaCas9 epigenetic screening platform. We evaluated dSaCas9 for targeted gene silencing in primary human T cells by conducting two high-throughput promoter tiling CRISPRi screens. Previous CRISPRi/a screens with dSpCas9 performed serial transductions with one lentivirus encoding the dCas9-effector and another lentivirus encoding for the gRNA-library[1]. To minimize the number of transduction events, we constructed an all-in-one CRISPRi lentiviral plasmid encoding for dSaCas9 fused to the KRAB repressor domain and a gRNA cassette (Extended Data Fig. 1a).

Next, we considered the protospacer adjacent motif (PAM) requirement for SaCas9. In the context of nuclease activity, SaCas9 is more active when targeting genomic regions upstream of the PAM (5' – NNGRRT-3') compared to the more relaxed PAM (5' – NNGRRV – 3'; where V = A, C, or G)[2]. However, several gRNA design tools do not require a thymine in the final position of the PAM[3, 4]. Moreover, the PAM preference for dSaCas9-based epigenetic effectors has not been rigorously characterized. To systematically evaluate this feature, we designed two independent gRNA libraries with the relaxed PAM variant (5'-NNGRRN-3') and tiled ~1,000 bp windows around the promoters of *CD2* and *B2M*. We chose *CD2* and *B2M* as gene targets because both are ubiquitously and high expressed genes encoding for surface markers and thus readily compatible with cell sorting-based screens. The CD2 and B2M gRNA libraries contained 141 and 217 targeting gRNAs, respectively, and 250 non-targeting gRNAs.

For each CRISPRi screen, we transduced primary human CD8+ T cells with the respective gRNA library and expanded the cells for 9-10 days before staining and sorting transduced cells in the lower and upper 10% tails of CD2 or B2M expression (Extended Data Fig. 1b). We recovered 16 and 5 targeting gRNAs enriched in the CD2 low and B2M low populations (Extended Data Fig. 1c and Extended Data Fig. 2a). Many enriched gRNAs were within an optimal window relative to the transcriptional start site (TSS) for gene silencing[4] (Extended Data Fig. 1d and Extended Data Fig. 2b). Although only a small fraction of the targeting gRNAs (11% of CD2 gRNAs and 2% of B2M gRNAs) were hits for each gene target, the gRNA hit rates (32% of CD2 gRNAs and 16% of B2M gRNAs)

were significantly higher for gRNAs targeting the strict PAM (5'-NNGRRT-3'). This is consistent with previous PAM characterization of SaCas9 for nuclease activity[2] and suggests that the thymine base in the final position of the PAM facilitates more efficient recognition and binding between dSaCas9 and the target DNA sequence (Extended Data Fig. 1e and Extended Data Fig. 2c).

Subsequent validation of CD2 and B2M gRNA screen hits revealed marked gene silencing and a wide range of activity across gRNAs, underscoring the unique capability of CRISPRi to tune gene expression levels (Extended Data Fig. 1f, Extended Data Fig. 2d,e, and Supplementary Fig. 1a,b). For example, the percentage of CD2 silenced cells varied from 7% to 89% depending on the gRNA (Extended Data Fig 1f and Supplementary Fig. 1a). The mean expression of CD2 in silenced cells was highly correlated with the percentage of silenced cells, indicating that the effect of a gRNA across a cell population is coupled to the magnitude of gRNA activity at a single cell level (Supplementary Fig. 1c). The most potent CD2 and B2M gRNAs targeted genomic sites adjacent to 5'-NNGRRT-'3 PAMs (Extended Data Fig. 1f and Extended Data Fig. 2d). As previously observed[5, 6], individual gRNA activity was strongly correlated with its fold-enrichment in the screen (Extended Data Fig. 1g and Supplementary Fig. 1d). Finally, we adapted this CRISPRi system for multiplex gene silencing by using a lentiviral plasmid with orthogonal mouse and human U6 promoters. We verified this system using the most potent CD2 and B2M gRNAs and only detected dual silenced cells when both CD2 and B2M gRNAs were delivered (Supplementary Fig. 1e-g).

Next, we developed efficient and compact dSaCas9-based activators using the small transactivation domain VP64. Using polyclonal Jurkat cell lines constitutively expressing dSaCas9 fused to either one copy of VP64 (dSaCas9^{VP64}) or two copies of VP64 (^{VP64}dSaCas9^{VP64}), we conducted parallel CRISPRa screens with a 400 gRNA library (306 *IL2RA* gRNAs and 94 non-targeting gRNAs) tiling a 5,000 bp window around the TSS of the transcriptionally silenced *IL2RA* gene (Extended Data Fig. 3a,b). Interestingly, there were three more gRNA hits in the ^{VP64}dSaCas9^{VP64} CRISPRa screen along with a shared set of five gRNA hits (Extended Data Fig. 1h,i). All gRNA hits targeted sites within a prominent open chromatin peak within 350 bp of the TSS with the majority located upstream of the TSS (Extended Data Fig. 3b). As with gene silencing, there was

a marked preference for 5'-NNGRRT-3' PAMs for gene activation with 75% of gRNA hits targeting this PAM variant (Extended Data Fig. 3c). Together, the relative gRNA position and PAM sequence were major predictors of gRNA efficacy, similar to the CRISPRi screen, as 24% (6/25) of 5'-NNGRRT-3' targeting IL2RA gRNAs within a ~1,000 bp window around the TSS were hits. Individual validation of all eight gRNA hits in both cell lines showed a significant increase in IL2RA expression with ^{VP64}dSaCas9^{VP64} consistently more potent than dSaCas9^{VP64} (Extended Data Fig. 1j,k and Extended Data Fig. 3d). Moreover, the most potent ^{VP64}dSaCas9^{VP64} gRNAs achieved equivalent levels of IL2RA gene activation as ^{VP64}dSpCas9^{VP64} paired with the best IL2RA gRNA from a published CRISPRa screen tiling the *IL2RA* locus in Jurkats[7] (Extended Data Fig. 1k).

Given the robust activity of ^{VP64}dSaCas9^{VP64} in Jurkat cells, we constructed an all-in-one CRISPRa lentiviral vector encoding for ^{VP64}dSaCas9^{VP64} and gRNA cassette for assays in primary human T cells. We hypothesized that the smaller size of dSaCas9 would lead to higher titer lentivirus than S. *pyogenes* Cas9 (SpCas9), thus reducing the quantity of T cells and reagents required to perform CRISPR-based screens with equivalent coverage. We tested this by transducing CD8+ T cells from two donors with serial titrations of all-in-one ^{VP64}dSaCas9^{VP64} and ^{VP64}dSpCas9^{VP64} lentiviruses. ^{VP64}dSaCas9^{VP64} produced nearly two-fold higher lentiviral titers than the equivalent ^{VP64}dSpCas9^{VP64} construct, thus requiring half the lentiviral volume to achieve the same transduction rate (Supplementary Fig. 2a-d). We then verified that ^{VP64}dSaCas9^{VP64} could potently activate endogenous gene expression of a transcriptionally silenced gene (*EGFR*) in primary human T cells (Supplementary Fig. 2e,f).

Supplementary Note 2: Design of TF gRNA library. We compiled a curated list of 110 TFs associated with T cell state and function based on motif enrichment in differentially accessible chromatin across T cell subsets[8-10,11] and manually appended the following 11 transcriptional and epigenetic regulators: *BACH2, TOX, TOX2, PRDM1, KLF2, BMI1, DNMT1, DNMT3A, DNMT3B, TET1,* and *TET2* for a total of 121 candidate genes (Supplementary Table 2). Based on our characterization of dSaCas9-based epigenome editors, we generated a gRNA library containing all specific, 5'-NNGRRT-3' PAM targeting gRNAs within a 1,000 bp window centered around the TSS of each gene. All genes were represented by at least 7 gRNAs with an average of 16 gRNAs per gene, except for PBX2 which did not have any gRNAs (Supplementary Fig. 3a,b). We added 120 non-targeting gRNAs as negative controls, bringing the final gRNA library to 2,099 gRNAs (Supplementary Table 2). We cloned the gRNA library into both all-in-one CRISPRi and CRISPRa lentiviral plasmids. Subsequent lentiviral titrations revealed a dose-dependent response to lentiviral volume with both CRISPRi and CRISPRa constructs eclipsing 90% transduction rates (Supplementary Fig. 3c).

Supplementary Note 3: Performing quality control of scRNA-seq data. To assess the quality and statistical power of our scRNA-seq data, we compared the quantity and magnitude of effects between targeting and non-targeting gRNAs. Targeting gRNAs were associated with significantly more differentially expressed genes (DEGs) and these gRNA-to-gene links had larger effect sizes than non-targeting gRNAs (Supplementary Fig. 6a-d). We therefore proceeded to evaluate the transcriptomic effects of silencing or activating each candidate gene.

Supplementary Note 4: Importance of gRNA coverage. We noticed that gRNA hits that did not validate in the scRNA-seq characterization were represented by fewer cells than validated gRNAs (Supplementary Fig. 7c). For example, the CRISPRa CREM-targeting gRNA that validated was represented by 635 cells, whereas the other CREM-targeting gRNA was represented only 142 cells and narrowly missed the significance threshold. Nevertheless, both gRNAs upregulated CREM and similarly affected gene expression programs (Supplementary Fig. 7d). Several BATF-targeting gRNAs were also underrepresented. To evaluate CREM and BATF gRNAs, we individually assayed a pair of CRISPRa gRNA hits targeting each gene and validated that each gRNA regulated CCR7 expression as predicted by the screen (Supplementary Fig. 7a,b). We suspect that several gRNAs were underrepresented in the scRNA-seq experiment due to gRNA-intrinsic features that either interfered with reverse transcription, oligo capture by the beads, or subsequent amplification. Underrepresented gRNAs were not depleted in the initial gRNA plasmid pool nor did we observe any fitness defects in individual validations. The same gRNAs were underrepresented in both CRISPRi and CRISPRa assays, further pointing to gene-independent effects.

Supplementary Note 5: MYB repression drives T cells towards an effector-like state. Mouse models of acute and chronic infection have implicated MYB as an essential positive regulator of stem-like memory CD8+ T cells[12] and a small and distinct CD62L+ precursor of exhausted T cell population[13]. In both contexts, MYB-deficient CD8+ T cells lacked therapeutic potential due to either impaired recall response or the inability to respond to checkpoint blockage. An important and lingering question has been whether MYB plays a similar role in human CD8+ T cells. Our scRNA-seq data revealed that MYB does indeed regulate human CD8+ T cell stemness with MYB silencing driving CD8+ T cells towards terminal effector T cells. MYB silencing led to downregulation of memory-associated TFs (*TCF7*, *KLF2*), lymph homing molecules (*CCR7*, *CD62L*, *S1PR1*), and cell-cycle inhibitors (*CDKN1B*). In addition, MYB-silenced cells had increased expression of effector-associated TFs (*TBX21*, *PRMD1*, *ZNF683*), effector molecules (*GZMB*, *PRF1*), inflammatory cytokines (*IFNG*, *TNF*), and positive cell-cycle regulators (*E2F1*, *CDC6*, *SKP2*, *CDC25A* and *KIF14*) (Extended Data Fig. 4a,b). The two MYB CRISPRi gRNAs were represented by the first and third most cells across both CRISPRi and CRISPRa screens, suggesting that MYB silencing promoted T cell proliferation (Fig. 2e).

Supplementary Note 6: NR1D1 activation synthetically programs a transcriptional profile with features of T cell exhaustion. *NR1D1* encodes a nuclear receptor subfamily 1 transcription factor and negatively regulates expression of core clock proteins that govern cyclical gene expression patterns. Integrative analysis of bulk ATAC-seq data across 12 independent studies of CD8 T cell dysfunction in cancer and infection found that the NR1D1 motif was enriched in open chromatin of exhausted T cells[11]. The causal role of NR1D1 in CD8 T cells, however, has not been studied. NR1D1 activation resulted in 646 upregulated and 293 downregulated genes (Extended Data Fig. 4d). In agreement with NR1D1 motif enrichment[11], a large set of effector and exhaustion-associated genes were markedly upregulated with NR1D1 activation. To better understand the magnitude of exhaustion induction by NR1D1, we calculated an exhaustion gene signature score using a defined set of 82 exhaustion-specific genes[14]. *NR1D1*-perturbed cells had a significantly higher exhaustion gene signature score than non-perturbed cells ((Extended Data Fig. 4e). Many memory-associated surface markers (*IL7R*, *CCR7*, *SELL*, *CD5*) and TFs (*TCF7*, *LEF1*) were downregulated, suggesting NR1D1 activation synthetically programs a transcriptional profile with features of T cell exhaustion.

Supplementary Note 7: Exploring kinetics of BATF3 expression with CRISPRa and ORF. To understand the kinetics and magnitude of BATF3 expression in human CD8+ cells, we performed a time course experiment where we transduced CD8+ T cells with control lentiviral vector (GFP or CRISPRa + NT gRNA), CRISPRa + BATF3 gRNA, or BATF3 open reading frame (ORF) and measured BATF3 transcript levels at multiple time points (Extended Data Fig. 5a). Consistent with other studies[15, 16], BATF3 expression spiked after T cell activation and tapered back to baseline levels by day 10 post-transduction. Both endogenous activation and ectopic BATF3 expression increased BATF3 levels relative to the controls, however, ectopic expression led to significantly higher levels of BATF3.

Supplementary Note 8: Chronic stimulation extensively remodels epigenetic landscape. Chronic antigen stimulation induced widespread changes in chromatin accessibility with 23,322 differentially accessible regions between acutely and chronically stimulated control cells. Many of these regions were proximal to memory and effector/exhaustion-genes (Supplementary Fig. 8).

Supplementary Note 9: Complex and highly specific transcription control mediated by AP-1 TFs. BATF3 is a member of the AP-1 TF family, which regulates diverse biological processes across many cell types through complex and highly specific transcriptional control. This transcriptional specificity is enabled by combinatorial interactions between AP-1 TFs, which form cell-type specific homo- or hetero-dimers to regulate distinct gene expression programs. Several AP-1 complexes such as BATF-JUN heterodimers can interact with interferon-regulatory factors (IRF) at AP-1-IRF consensus elements, providing further flexibility in gene regulation[17].

Supplementary Methods

Supplementary Methods 1: More detailed protocol of transfections for high-titer lentiviral production. 1.2 x 10^6 or 7 x 10^6 HEK293T cells were plated in a 6 well plate or 10 cm dish in the afternoon with 2 mL or 12 mL of complete opti-MEM (Opti-MEM™ I Reduced Serum Medium supplemented with 1x Glutamax, 5% FBS, 1 mM Sodium Pyruvate, and 1x MEM Non-Essential Amino Acids). The next morning, HEK293T cells were transfected with 0.5 µg pMD2.G, 1.5 µg psPAX2, and 0.5 µg transgene for 6 well transfections or 3.25 µg pMD2.G, 9.75 µg psPAX2, and 4.3 µg transgene for 10 cm dishes using Lipofectamine 3000. Media was exchanged 6 hours after transfection and lentiviral supernatant was collected and pooled at 24 hours and 48 hours after transfection.

Supplementary Methods 2: HOMER motif analyses. We defined the set of target differentially accessible peaks using DESeq2 ($P_{adj} < 0.05$) and a background set of non-dynamic regions (p value > 0.2 and $|\log_2(\text{fold change})| < 0.2$) with all sets having a sufficiently large number of sequences. Next, for each set we extracted FASTA sequences from the human reference genome (GRCh38) and ran findMotif.pl to discover motifs and compute the enrichment over background. By default, this function uses a hypergeometric distribution to score motifs to calculate enrichment p-values, controlling for differences in GC-content across target and background sets.

Supplementary Methods 3: Gating strategy for viable tumor cells in in vitro co-culture experiment. We quantified the percentage of viable tumor cells using the following strict gating strategy. First, T cells were excluded based on cell size and GFP signal. Next, a gate was set around the double negative (FVD-, Annexin V-) fraction containing viable tumor cells and cellular debris. Visualizing these events on SSC vs. FSC, a gate was set to encompass events located in the bottom left quadrant. This gate was then inverted to exclude debris from the viability calculation and moved immediately beneath the T cell exclusion gate on the gating hierarchy. Tumor viability was reported using the percentage of tumor cells in the final double negative (FVD-, Annexin V-) gate.

Supplementary Methods 4: Flow cytometry analysis of input and tumor infiltrating CAR T cells. Fluorophore conjugated antibodies against the following antigens were used for input and day 3 cells (All Biolegend unless otherwise noted): panel 1: myc-APC (Cell Signaling Technologies), CD3-BUV737 and CD8-BUV395 (BD Biosciences), TIGIT-BV605, LAG3-BV786, CD127-PERCPCy5.5, PD1-BV711, Tim3-PECy5, GranzymeB-PECy7, TCF1-BV421, Ki67-BV510, and IFN-g; panel 2: myc-APC, CD3-BUV737, CD8-BUV395, CD39-PECF594, CD56-BV605, CD45RO-BV786, CD45RA-PEcy5, CD28-PECy7, CCR7-BV711, CD62L-BV510, CTLA4-BV421, Tbet-PERCPCy5.5, EOMEs-PE. For day 19 post CAR T cell delivery analyses anti-human CD45-FITC (Biolegend) staining was added to the above panels to increase sensitivity of CAR T cell detection, as we anticipated reduction in numbers, and the following additional panel was added against the following antigens: CD45-FITC, myc-APC, CD3-BUV737, CD8-BUV395, LAG3-BV786, TIM3-PECy5, CXCR3-BV711, CD4-BV510, TNF-BV605, ID2-PECy7, GATA3-BV421, IRF4-PERCPCy5.5, ID3-PE.

References

1. Schmidt, R. et al. CRISPR activation and interference screens decode stimulation responses in primary human T cells. *Science (New York, N.Y.)* **375** (2022).
2. Ran, F.A. et al. In vivo genome editing using Staphylococcus aureus Cas9. *Nature* **520**, 186-191 (2015).
3. Doench, J.G. et al. Optimized sgRNA design to maximize activity and minimize off-target effects of CRISPR-Cas9. *Nature biotechnology* **34**, 184-191 (2016).
4. Sanson, K.R. et al. Optimized libraries for CRISPR-Cas9 genetic screens with multiple modalities. *Nat Commun* **9**, 5416 (2018).
5. Klann, T.S. et al. CRISPR-Cas9 epigenome editing enables high-throughput screening for functional regulatory elements in the human genome. *Nature biotechnology* **35**, 561-568 (2017).
6. Black, J.B. et al. Master Regulators and Cofactors of Human Neuronal Cell Fate Specification Identified by CRISPR Gene Activation Screens. *Cell reports* **33**, 108460 (2020).
7. Simeonov, D.R. et al. Discovery of stimulation-responsive immune enhancers with CRISPR activation. *Nature* **549**, 111-115 (2017).
8. Philip, M. et al. Chromatin states define tumour-specific T cell dysfunction and reprogramming. *Nature* **545**, 452-456 (2017).
9. Krishna, S. et al. Stem-like CD8 T cells mediate response of adoptive cell immunotherapy against human cancer. *Science (New York, N.Y.)* **370**, 1328-1334 (2020).
10. Galletti, G. et al. Two subsets of stem-like CD8+ memory T cell progenitors with distinct fate commitments in humans. *Nature Immunology* (2020).
11. Pritykin, Y. et al. A unified atlas of CD8 T cell dysfunctional states in cancer and infection. *Mol Cell* **81**, 2477-2493 e2410 (2021).
12. Gautam, S. et al. The transcription factor c-Myb regulates CD8+ T cell stemness and antitumor immunity. *Nature Immunology* **20**, 337-349 (2019).
13. Tsui, C. et al. MYB orchestrates T cell exhaustion and response to checkpoint inhibition. *Nature* **609** (2022).
14. Zheng, C. et al. Landscape of Infiltrating T Cells in Liver Cancer Revealed by Single-Cell Sequencing. *Cell* **169**, 1342-1356 (2017).
15. Ataide, M.A. et al. BATF3 programs CD8(+) T cell memory. *Nat Immunol* **21**, 1397-1407 (2020).
16. Calderon, D. et al. Landscape of stimulation-responsive chromatin across diverse human immune cells. *Nature genetics* **51**, 1494-1505 (2019).
17. Murphy, T.L., Tussiwand, R. & Murphy, K.M. Specificity through cooperation: BATF-IRF interactions control immune-regulatory networks. *Nat Rev Immunol* **13**, 499-509 (2013).
