# Supplementary material for: Transcriptional and epigenetic regulators of human CD8+ T cell function identified through orthogonal CRISPR screens
Source: Nat Genet. 2023 Nov 9;55(12):2211–23. doi: 10.1038/s41588-023-01554-0 (PMC10703699; doi:10.1038/s41588-023-01554-0)
Supplement: Supplementary file 2 — Reporting Summary [file 41588_2023_1554_MOESM2_ESM.pdf]

nature portfolio

Corresponding author(s): Charles Gersbach

Last updated by author(s): Dec 4, 2022 Sep 1, 2013

Reporting Summary

Nature Portfolio wishes to improve the reproducibility of the work that we publish. This form provides structure for consistency and transparency in reporting. For further information on Nature Portfolio policies, see our Editorial Policies and the Editorial Policy Checklist.

Statistics

For all statistical analyses, confirm that the following items are present in the figure legend, table legend, main text, or Methods section.

n/a	Confirmed	
☐	☒	The exact sample size (*n*) for each experimental group/condition, given as a discrete number and unit of measurement
☐	☒	A statement on whether measurements were taken from distinct samples or whether the same sample was measured repeatedly
☐	☒	The statistical test(s) used AND whether they are one- or two-sided<br>*Only common tests should be described solely by name; describe more complex techniques in the Methods section.*
☐	☒	A description of all covariates tested
☐	☒	A description of any assumptions or corrections, such as tests of normality and adjustment for multiple comparisons
☐	☒	A full description of the statistical parameters including central tendency (e.g. means) or other basic estimates (e.g. regression coefficient) AND variation (e.g. standard deviation) or associated estimates of uncertainty (e.g. confidence intervals)
☐	☒	For null hypothesis testing, the test statistic (e.g. *F*, *t*, *r*) with confidence intervals, effect sizes, degrees of freedom and *P* value noted<br>*Give P values as exact values whenever suitable.*
☒	☐	For Bayesian analysis, information on the choice of priors and Markov chain Monte Carlo settings
☒	☐	For hierarchical and complex designs, identification of the appropriate level for tests and full reporting of outcomes
☐	☒	Estimates of effect sizes (e.g. Cohen's *d*, Pearson's *r*), indicating how they were calculated

Our web collection on statistics for biologists contains articles on many of the points above.

Software and code

Policy information about availability of computer code

Data collection	Sony SH800 Sorter and Fortessa X 20 were used for cell analysis and sorting<br>Bio-RAD CFX96 Real Time PCR Detection System was used for qPCR<br>Illumina MiSeq, Illumina NextSeq 2000, and Illumina NovaSeq 6000 were used for next-generation sequencing<br>Thermo Nanodrop and Qubit Fluorometer were used for DNA and RNA quantification
Data analysis	Graphpad Prism v.9.0.2 - graphs and statistics<br>R v4.2.l - data analysis and visualization<br>Python v3.7.6 - data visualization<br>FlowJo vl0.8.1 - flow cytometry analysis<br>Bowtie2 v2.3.5.l and DESeq2 vl.36.0 - gRNA enrichment for flow-based CRISPR screens<br>Seurat v4.l.0 - single cell RNA-seq analysis<br>Seurat v4.2.0 - single cell RNA-seq analysis<br>Trimmomatic v0.32, STAR v.2.4.la, featureCounts in subread package vl.4.6-p4, and DESeq2 vl.36.0 were used for RNA-seq analyses<br>FastQC v0.11.2, Trimmomatic v0.32, Bowtie vl.0.0, bedtools2 v2.25.0, Picard MarkDuplicates vl.130, deeptools bamCoverage v3.0.l, MACS2 v2.l.l.20160309, featureCounts in subread package vl.4.6-p4, DESeq2 vl.36.0, ChIPseeker vl.32.0, and Homer v4.11 were used for ATAC-seq analyses

MAGeck v.0.5.9.4 - gene-level enrichment for flow-based CRISPRko screens

For manuscripts utilizing custom algorithms or software that are central to the research but not yet described in published literature, software must be made available to editors and reviewers. We strongly encourage code deposition in a community repository (e.g. GitHub). See the Nature Portfolio guidelines for submitting code & software for further information.

Data

Policy information about availability of data

All manuscripts must include a data availability statement. This statement should provide the following information, where applicable:

- Accession codes, unique identifiers, or web links for publicly available datasets
- A description of any restrictions on data availability
- For clinical datasets or third party data, please ensure that the statement adheres to our policy

All data associated with this study are present in the manuscript or its Supplementary Information files. GRCh38 reference genome was used for gRNA library designs and alignments. All CRISPR screening, scRNA-seq, RNA-seq, and ATAC-seq data have been deposited in the Gene Expression Omnibus (GEO) under accession number: GSE218988.

Human research participants

Policy information about studies involving human research participants and Sex and Gender in Research.

Reporting on sex and gender	N/A
Population characteristics	N/A
Recruitment	N/A
Ethics oversight	N/A

Note that full information on the approval of the study protocol must also be provided in the manuscript.

Field-specific reporting

Please select the one below that is the best fit for your research. If you are not sure, read the appropriate sections before making your selection.

☒ Life sciences ☐ Behavioural & social sciences ☐ Ecological, evolutionary & environmental sciences

For a reference copy of the document with all sections, see nature.com/documents/nr-reporting-summary-flat.pdf

Life sciences study design

All studies must disclose on these points even when the disclosure is negative.

Sample size	No statistical methods were used to predetermine sample sizes. For CRISPR-based assays, sample sizes (n = 2 or n =3) were chosen to be in line with many published CRISPR-based screens and screen validation in primary T cells (PMIDs: 30449619, 35113687, 35817986, 35750052, 36356142). For in vivo studies, samples sizes (n = 4 or n = 5 mice per treatment) were chosen to be in line with other published reports of in vivo tumor control (PMIDs: 36002574, 31802004)
Data exclusions	No data were excluded.
Replication	All experiments have been replicated successfully with at least two independent biological replicates. For BATF3 overexpression studies, all orthogonal assays (flow cytometry, RNA-seq, ATAC-seq, in vitro and in vivo tumor killing) were performed in T cells from unique donors, which corroborated the findings from each individual assay and confirmed the effects of BATF3 are donor-independent.
Randomization	For in vitro assays, each T cell donor was treated with both control and experimental conditions, so randomization was not necessary as all assays were donor-matched. For in vivo studies, tumor bearing mice were randomly assigned into the following treatment groups: untreated, standard CART cells, BATF3 CART cells to ensure there was no biases in assigning mice to a specific treatment group.
Blinding	No blinding was involved in this study. All in vitro assays involved either equipment-based quantitative measurements or sequencing data rather than subjective rating of data that could be affected by no blinding. For in vivo studies, mice were randomly grouped into treatment groups. Blinding was not necessary for group allocation because the range of tumor volumes was narrow across mice. As tumor volume size is a quantitative measurement rather than a subjective rating of mouse health, blinding was not necessary for tumor measurements.

Reporting for specific materials, systems and methods

We require information from authors about some types of materials, experimental systems and methods used in many studies. Here, indicate whether each material, system or method listed is relevant to your study. If you are not sure if a list item applies to your research, read the appropriate section before selecting a response.

Materials & experimental systems

n/a	Involved in the study
☐	☒ Antibodies
☐	☒ Eukaryotic cell lines
☒	☐ Palaeontology and archaeology
☐	☒ Animals and other organisms
☒	☐ Clinical data
☒	☐ Dual use research of concern

Methods

n/a	Involved in the study
☒	☐ ChIP-seq
☐	☒ Flow cytometry
☒	☐ MRI-based neuroimaging

Antibodies

Antibodies used

This information can also be accessed in Supplementary Table 5.
Manufacturer Antibody Target Fluorophore/Sequence Clone Catalog #
Thermo CD2 PE RPA-2.10 12-0029-42
Biolegend B2M PE A17082A 395704
Thermo IL2RA PE-Cy7 BC96 25-0259-42
BD Biosciences EGFR bv-421 EGFR.1 742602
BD Biosciences CCR7 FITC 150503 561271
BD Biosciences CD8 bv-421 HIT8a 740078
BD Biosciences IL7RA PE HIL-7R-M21 557938
Thermo LAG3 PE 3DS223H 12-2239-42
Biolegend TIM3 PE-Cy5 F38-2E2 345052
Thermo TIGIT PerCP-eFluor710 MBSA43 46-9500-42
BD Biosciences PD1 PE-Cy7 EH12.1 561272
Cell Signaling Technology Myc-tag Alexa Fluor 647 9B11 2233S
StemCell Technologies Thy1.1 PE OX-7 60024PE
Biolegend CD2 TACGATTTGTCAGGG TS1/8 309231
Biolegend Anti-human Hashtag 1 GTCAACTCTTTAGCG LNH-94 and 2M2 394661
Biolegend Anti-human Hashtag 2 TGATGGCCTATTGGG LNH-94 and 2M2 394663
Biolegend Anti-human Hashtag 3 TTCCGCCTCTCTTTG LNH-94 and 2M2 394665
Biolegend Anti-human Hashtag 4 AGTAAGTTCAGCGTA LNH-94 and 2M2 394667
Biolegend Anti-human Hashtag 5 AAGTATCGTTTCGCA LNH-94 and 2M2 394669
Biolegend Anti-human Hashtag 6 GGTTGCCAGATGTCA LNH-94 and 2M2 394671
Biolegend Anti-human Hashtag 7 TGTCTTTCCTGCCAG LNH-94 and 2M2 394673
Biolegend Anti-human Hashtag 8 CTCCTCTGCAATTAC LNH-94 and 2M2 394675

Validation

We either used the manufacturer recommended antibody dilution or titrated the antibodies ourselves to determine the dilution that achieved the optimal signal to background ratio. We used the following dilutions of each antibody.
Antibody Target Fluorophore/Sequence Dilution
CD2 PE 1:50
B2M PE 1:50
IL2RA PE-Cy7 1:50
EGFR bv-421 1:50
CCR7 FITC 1:100
CD8 bv-421 1:50
IL7RA PE 1:100
LAG3 PE 1:50
TIM3 PE-Cy5 1:50
TIGIT PerCP-eFluor710 1:50
PD1 PE-Cy7 1:100
Myc-tag Alexa Fluor 647 1:50
Thy1.1 PE 1:300
CD2 TACGATTTGTCAGGG 1:12.5
Anti-human Hashtag 1 GTCAACTCTTTAGCG 1:50
Anti-human Hashtag 2 TGATGGCCTATTGGG 1:50
Anti-human Hashtag 3 TTCCGCCTCTCTTTG 1:50
Anti-human Hashtag 4 AGTAAGTTCAGCGTA 1:50
Anti-human Hashtag 5 AAGTATCGTTTCGCA 1:50
Anti-human Hashtag 6 GGTTGCCAGATGTCA 1:50
Anti-human Hashtag 7 TGTCTTTCCTGCCAG 1:50
Anti-human Hashtag 8 CTCCTCTGCAATTAC 1:50

Eukaryotic cell lines

Policy information about cell lines and Sex and Gender in Research

Cell line source(s)	HEK293Ts, Jurkats, SKBR3s, and HCC1954s were from ATCC. Pooled PBMCs were from ZenBio. Individual CD8 T cell donors were from StemCell Technologies.
Authentication	Cell lines were authenticated by ATCC using STR profiling. Primary CD8 T cells were authenticated by the vendor for quantity and purity. We then independently authenticated all primary human CD8 T cells using flow cytometry.
Mycoplasma contamination	Cell lines were not tested for mycoplasma contamination.
Commonly misidentified lines (See ICLAC register)	No commonly misidentified lines were used in this study.

Animals and other research organisms

Policy information about studies involving animals; ARRIVE guidelines recommended for reporting animal research, and Sex and Gender in Research

Laboratory animals	6–8-week-old female immunodeficient NOD/SCID gamma (NSG) mice were obtained from Jackson Laboratory and then housed in 12 hours light/dark cycles, at an ambient temperature (21 +/- 3°C) with relative humidity (50 +/- 20%) and handled in pathogen-free conditions
Wild animals	No wild animals were used in these studies.
Reporting on sex	Only female mice were used for in vivo tumor killing studies because we were using an orthotopic breast cancer model where tumor cells were implanted into the mammary pad of NSG mice.
Field-collected samples	There were no field-collected samples used in this study.
Ethics oversight	All experiments involving animals were conducted with strict adherence to the guidelines for the care and use of laboratory animals of the National Institutes of Health (NIH). All experiments were approved by the Institutional Animal Care and Use Committee (IACUC) at Duke University (protocol number AB0-22-07).

Note that full information on the approval of the study protocol must also be provided in the manuscript.

Flow Cytometry

Plots

Confirm that:

☒ The axis labels state the marker and fluorochrome used (e.g. CD4-FITC).

☒ The axis scales are clearly visible. Include numbers along axes only for bottom left plot of group (a 'group' is an analysis of identical markers).

☒ All plots are contour plots with outliers or pseudocolor plots.

☒ A numerical value for number of cells or percentage (with statistics) is provided.

Methodology

Sample preparation	Cells were spun down at 300xg for 5 minutes, resuspended in flow buffer (lx PBS with 0.5% BSA and 2mM EDTA), stained with the appropriate antibodies for 30 minutes, washed with flow buffer, and resuspended in flow buffer for flow cytometry analysis or sorting.
Instrument	An SH800 FACS Cell Sorter (Sony Biotechnology) or Fortessa X 20 were used for cell sorting and analysis.
Software	FlowJo vl0.8.1 was used to analyze all flow cytometry data.
Cell population abundance	We collected around 10,000-30,000 live cells for the final analysis for all experiments except for the in vivo TIL characterization studies, where we analyzed around 200 - 10,000 live cells per mouse.
Gating strategy	We used the following general gating strategy for all flow cytometry data:<br>1. Cell population was gated using SSC-A vs FSC-A<br>2. Viable, singlets (FSC-H vs FSC-A) were gated<br>3. Transduced cells (when applicable) were gated using FSC-H vs. the specific channel that detected the marker (e.g. GFP, Thy1.1) for transduction. This gate was set using an unstained or FMO control.<br>4. Cells positive for the markers of interest were gated (gate was defined using an FMO) using FSC-H vs. appropriate channel.

☒ Tick this box to confirm that a figure exemplifying the gating strategy is provided in the Supplementary Information.
